# From Chalcogen Bonding to S–π Interactions in Hybrid Perovskite Photovoltaics

**DOI:** 10.1002/advs.202405622

**Published:** 2024-07-03

**Authors:** Weifan Luo, SunJu Kim, Nikolaos Lempesis, Lena Merten, Ekaterina Kneschaurek, Mathias Dankl, Virginia Carnevali, Lorenzo Agosta, Vladislav Slama, Zachary VanOrman, Miłosz Siczek, Wojciech Bury, Benjamin Gallant, Dominik J. Kubicki, Michal Zalibera, Laura Piveteau, Marielle Deconinck, L. Andrés Guerrero‐León, Aaron T. Frei, Patricia A. Gaina, Eva Carteau, Paul Zimmermann, Alexander Hinderhofer, Frank Schreiber, Jacques‐E. Moser, Yana Vaynzof, Sascha Feldmann, Ji‐Youn Seo, Ursula Rothlisberger, Jovana V. Milić

**Affiliations:** ^1^ Adolphe Merkle Institute University of Fribourg Fribourg 1700 Switzerland; ^2^ Department of Nanoenergy Engineering Pusan National University Busan 46241 South Korea; ^3^ Laboratory of Computational Chemistry and Biochemistry Institute of Chemical Sciences and Engineering École Polytechnique Fédérale de Lausanne Lausanne 1015 Switzerland; ^4^ Institute of Applied Physics University of Tübingen 72076 Tübingen Germany; ^5^ Rowland Institute Harvard University Cambridge MA 02142 USA; ^6^ Faculty of Chemistry University of Wrocław Wrocław 50–383 Poland; ^7^ School of Chemistry University of Birmingham Birmingham B15 2TT UK; ^8^ Institute of Physical Chemistry and Chemical Physics Slovak University of Technology Bratislava 81237 Slovakia; ^9^ Laboratory of Magnetic Resonance EPFL Lausanne 1015 Switzerland; ^10^ Chair for Emerging Electronic Technologies Technical University of Dresden 02062 Dresden Germany; ^11^ Leibniz Institute for Solid State and Materials Research Dresden Dresden University of Technology Helmholtzstraße 20 01069 Dresden Germany; ^12^ Photochemical Dynamic Group Institute of Chemical Sciences and Engineering École Polytechnique Fédérale de Lausanne Lausanne 1015 Switzerland

**Keywords:** chalcogen bonding, layered perovskites, low‐dimensional perovskites, photovoltaics, supramolecular engineering

## Abstract

The stability of hybrid organic–inorganic halide perovskite semiconductors remains a significant obstacle to their application in photovoltaics. To this end, the use of low‐dimensional (LD) perovskites, which incorporate hydrophobic organic moieties, provides an effective strategy to improve their stability, yet often at the expense of their performance. To address this limitation, supramolecular engineering of noncovalent interactions between organic and inorganic components has shown potential by relying on hydrogen bonding and conventional van der Waals interactions. Here, the capacity to access novel LD perovskite structures that uniquely assemble through unorthodox S‐mediated interactions is explored by incorporating benzothiadiazole‐based moieties. The formation of S‐mediated LD structures is demonstrated, including one‐dimensional (1D) and layered two‐dimensional (2D) perovskite phases assembled via chalcogen bonding and S–π interactions. This involved a combination of techniques, such as single crystal and thin film X‐ray diffraction, as well as solid‐state NMR spectroscopy, complemented by molecular dynamics simulations, density functional theory calculations, and optoelectronic characterization, revealing superior conductivities of S‐mediated LD perovskites. The resulting materials are applied in n‐i‐p and p‐i‐n perovskite solar cells, demonstrating enhancements in performance and operational stability that reveal a versatile supramolecular strategy in photovoltaics.

## Graphical abstract

A new generation of low‐dimensional hybrid halide perovskite materials assembled via chalcogen bonding and S–π interactions is demonstrated by a combination of techniques, including X‐ray diffraction and solid‐state nuclear magnetic resonance spectroscopy, complemented by molecular dynamics simulations, density functional theory calculations, and optoelectronic characterization, revealing superior conductivities and enhancements in performance and operational stabilities in perovskite solar cells.

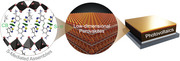

## 1 Introduction

**Figure 1:**
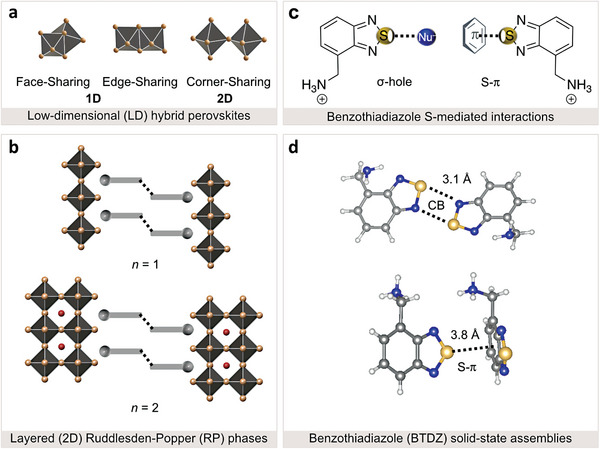
Low‐dimensional (LD) S‐mediated hybrid perovskite structures. a) Schematic representation of octahedral connectivity in LD perovskites based on face‐ and edge‐sharing 1D and corner‐sharing layered (2D) structures. b) Structural representation of Ruddlesden–Popper layered (2D) perovskite phases based on *S*_2_A_n‐1_M_n_X_3n+1_ formulations for *n* = 1 (top) and *n* = 2 (bottom) compositions with spacers (*S*) as grey rods, A cations as orange spheres (A = FA), halide anions as yellow spheres (X = I), and metal cations (M = Pb) forming the corner‐sharing {MX_6_} octahedral framework. c) Structure of benzo[*c*][1,2,5]thiadiazol‐4‐ylmethanammonium (BTDZ) spacer with the representation of the σ‐hole interaction between sulfur (S) and a nucleophilic acceptor (Nu^−^) associated with chalcogen‐bonding (CB, left) and S–π interactions (right). d) Schematic of solid‐state assemblies of BTDZ dimers mediated by CB (top) and S–π interactions (bottom) with the binding lengths based on the corresponding crystal structures of (BTDZ)I (top) and (BTDZ)_2_PbBr_4_ (bottom; CCDC 2342700–2342702, further details are provided in the Supporting Information).

Hybrid organic–inorganic metal halide perovskite materials have emerged as a promising alternative to traditional semiconductors in new‐generation solar cells due to their low cost, modular structure, and optoelectronic properties that offer exceptional photovoltaic performances.^[^ ^1^ ^]^ However, their long‐term operational stability remains a challenge to practical applications.^[^ ^2^ ^]^ The stability of perovskite solar cells is affected by several factors, including moisture and ion migration, which is accelerated by temperature changes, irradiation, and voltage bias.^[^ ^3^, ^4^, ^5^, ^6^ ^]^ Controlling these factors to achieve competitive efficiency and long‐term stability has become an essential prerequisite for successful applications of perovskite solar cells. To this end, low‐dimensional (LD) perovskites that incorporate hydrophobic organic moieties between perovskite slabs provide a strategy for enhanced operational stability, increasing resilience against moisture and ion migration.^[^ ^7^, ^8^, ^9^, ^10^ ^]^ This has involved both edge‐ and face‐sharing one‐dimensional (1D; **Figure** **1**a) and corner‐sharing layered (2D) octahedral frameworks (Figure 1b).^[^ ^11^ ^]^ Despite these advantages, their performance remains inferior to 3D perovskites due to poor charge transport,^[^ ^12^, ^13^ ^]^ which requires controlling LD perovskite assemblies and their (opto)electronic properties.^[^ ^14^, ^15^ ^]^ The structure of LD perovskites involves organic moieties as spacers (*S*) templating 3D perovskite slabs (Figure 1a), which are defined by the AMX_3_ formula, representing a corner‐sharing {MX_6_} octahedral framework, where A is a central cation (typically methylammonium (MA^+^), formamidinium (FA^+^), or Cs^+^) within the cuboctahedral cavity, M is a divalent metal ion (such as Pb^2+^), and X a halide anion (Cl^−^, Br^−^, I^−^).^[^ ^16^, ^17^, ^18^, ^19^ ^]^ Unlike 1D edge‐ or face‐sharing *S*MX_3_ “perovskitoid” structures,^[^ ^11^ ^]^ corner‐sharing 2D perovskites are typically based on the *S*_x_A_n‐1_M_n_X_3n+1_ composition incorporating monofunctional (x = 2) or bifunctional (x = 1) alkylammonium‐functionalized spacers (*S*).^[^ ^17^, ^18^, ^19^, ^20^ ^]^ They commonly form two types of 2D phases, namely the Ruddlesden–Popper (RP) and the Dion–Jacobson (DJ) phases.^[^ ^17^, ^18^, ^19^, ^20^ ^]^ The RP phases are based on *S*_2_A_n‐1_M_n_X_3n+1_ compositions incorporating mostly monofunctional spacers (*S*) with a half‐unit‐cell displacement between the adjacent slabs (Figure 1b).^[^ ^17^, ^21^ ^]^ On the contrary, DJ systems are typically based on the *S*A_n‐1_M_n_X_3n+1_ formula involving bifunctional spacers (*S*) within well aligned slabs.^[^ ^16^ ^]^ Since most spacers are electronically insulating, these materials act as quantum wells with charges confined to the inorganic slabs, determining their optoelectronic properties depending on the number of inorganic layers (*n*).^[^ ^20^ ^]^ The capacity to tailor their properties as a function of composition and interplay of interactions is an essential factor defining their application.^[^ ^14^, ^15^ ^]^

Noncovalent (i.e., supramolecular) interactions play a critical role in the structural and optoelectronic characteristics of hybrid LD perovskites.^[^ ^15^ ^]^ While this has so far primarily involved hydrogen bonding and van der Waals^[^ ^7^, ^15^, ^22^ ^]^ or π‐based interactions,^[^ ^23^, ^24^, ^25^ ^]^ other unorthodox interactions^[^ ^15^, ^26^ ^]^ are unexplored despite their potential to enhance the functionality of hybrid perovskite materials.^[^ ^15^, ^27^, ^28^ ^]^ In particular, this refers to chalcogen‐mediated interactions, such as chalcogen (Ch)–π interactions^[^ ^29^, ^30^, ^31^ ^]^ or σ–hole chalcogen bonding (CB) between a polarized Ch atom and a π system or a Lewis base, respectively, similar to halogen bonding,^[^ ^26^, ^27^, ^28^ ^]^ which remains unexplored in halide perovskite materials and devices to date. The electrostatic attraction between CB donors and nucleophilic acceptors originates from the anisotropic distribution of electron density around the chalcogen atom, resulting in the formation of a region of relatively positive electrostatic potential, known as the *σ‐hole* (Figure 1b).^[^ ^29^, ^30^ ^]^ While Ch‐mediated interactions have been shown to enable the control of structural and optoelectronic characteristics of functional materials and networks,^[^ ^31^, ^32^ ^]^ their application in perovskite photovoltaics is yet to be established.

Here, we report S‐mediated LD perovskite structures incorporating functionalized benzothiadiazole‐based spacers,^[^ ^29^, ^30^ ^]^ namely benzo[*c*][1,2,5]thiadiazol‐4‐ylmethylammonium (BTDZ; Figure 1c) that uniquely assemble through CB and S–π interactions (Figure 1d). The formation of S‐ mediated LD perovskites is evidenced by X‐ray diffraction and solid‐state NMR spectroscopy, complemented by theoretical insights into the nature of S‐mediated assemblies, including CB and S–π interactions. Furthermore, optoelectronic characterization provides insights into the interplay of interactions between organic and inorganic components through a combination of spectroscopic techniques, revealing superior conductivity of the BTDZ‐based perovskites with an appropriate energy level alignment. Finally, BTDZ‐based perovskites were applied at the interface with the charge‐transport layers in conventional (n‐i‐p and p‐i‐n) perovskite solar cells, demonstrating improvements in operational stability without compromising their photovoltaic performance.

## 2 Results and Discussion

Functionalized benzo[*c*][1,2,5]thiadiazol‐4‐methylammonium (BTDZ) halide (X = I, Br) spacer moieties were designed to enable their assembly via chalcogen bonding (CB; Figure 1c).^[^ ^32^ ^]^ This involves a benzothiadiazole core featuring CB interactions through “2S–2N” squares,^[^ ^32^ ^]^ as apparent in the corresponding crystal structures of the organic spacer dimers (Figure 1d). This is relevant for 2D RP phases that feature a van der Waals gap in the organic layer, which could be tailored through (supra)molecular engineering.^[^ ^15^ ^]^ Moreover, the BTDZ core was functionalized by methylammonium groups to facilitate the binding to the inorganic framework in the formation of LD perovskite structures (Figure 1c,d).^[^ ^11^, ^22^, ^33^ ^]^ The capacity to form chalcogen bonds in the BTDZ‐based layered perovskite frameworks was theoretically assessed for their feasibility.

### 2.1 Supramolecular Design

Benzothiadiazole‐based layered hybrid perovskite materials of (BTDZ)_2_PbI_4_ (*n* = 1) composition were analyzed by classical molecular dynamics (CMD) simulations to assess the preferred structural arrangements, without any assumption on the preferred orientation of the organic moieties in the layer. Moreover, ab initio molecular dynamics (AIMD) simulations complemented by density functional theory (DFT) calculations were used for quantification of the energetic ordering of the statistically prevalent interaction pattern and for visual representation of interactions (simulation details are provided in Figure S1–S8, and Table S1, Supporting Information).

**Figure 2:**
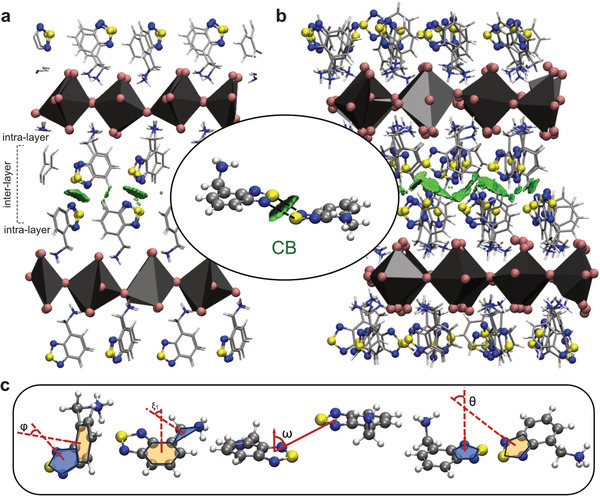
Theoretical analysis of the formation of CB in an *n* = 1 RP model system of (BTDZ)_2_PbI_4_. Snapshot of the *parallel in‐plane* configuration minimized at a) 0 K via DFT simulations and b) 300 K and 1 atm via AIMD simulations. The inset highlights a statistically dominant BTDZ interaction motif for the formation of interlayer CB interactions indicated with black broken lines. CB interactions are illustrated by green isosurfaces (as generated by NCIPLOT, detailed in the Methods section and further discussion). c) Schematic representations of the four angles used to describe the conformations (detailed in Figure S3, Supporting Information). The comparison with the experimental data is further discussed below.

For this purpose, we first investigated the relative stability of different molecular arrangements on the ab initio level for an *n* = 1 RP (BTDZ)_2_PbI_4_ model system, and we considered both initial molecular conformations with and without CB (Figure S1, Supporting Information). In the first configuration, denoted as parallel in‐plane, BTDZ molecules of the two adjacent organic layers (i.e., inter‐layer, **Figure** **2**) were fully aligned and in‐plane, comparable to the (BTDZ)I crystal structure (Figure 1d, top). In the second configuration, hereafter called rotated interlayer, BTDZ molecules of the two adjacent organic layers were at an angle to each other while parallel within the same layer (Figure 2a). In the third configuration, termed rotated intralayer, BTDZ molecules were at an angle both within the same organic layer (i.e., intralayer) and to the neighboring layer (i.e., interlayer). Finally, in the last, parallel displaced configuration, BTDZ in each layer are parallel to one another yet displaced to their counterparts in the neighboring layer. At 0 K, DFT simulations identified the rotated interlayer configuration as the energetically most favorable, followed closely by the rotated intralayer arrangement with an energy difference of only 0.007 eV per unit cell. The parallel in‐plane and parallel displaced configurations appeared to be isoenergetic, exhibiting an energy difference of 0.06 eV per unit cell above the rotated intralayer. In each of the four configurations, the asymmetric structure of BTDZ renders the nitrogen atoms of the thiadiazol core inequivalent (labeled as N_1_ and N_2_), resulting in three possible interaction patterns between BTDZ molecules that allow for CB (Figure S2, Supporting Information). Upon further analysis of these interaction patterns across configurations, the CB for the most stable rotated intralayer configuration was first identified at 0 K (Figure 2a), as visualized by using NCIPLOT based on the calculated ab initio electron density and its reduced density gradient upon interaction.^[^ ^34^ ^]^ To assess the thermal stability of CB interactions, the four system arrangements considered above were used as initial configurations in AIMD simulations at 300 K and 1 atm. In agreement with the DFT energetics at 0 K, the rotated interlayer system was identified as energetically most favorable at 300 K by 0.03 eV per unit cell with respect to the less stable rotated intralayer followed by the parallel in‐plane and parallel displaced configurations, which were isoenergetic, with an energy difference of 0.06 eV per unit cell above the most stable one. These energy values were calculated after time‐averaging the potential energies over the last 4 ps of each AIMD trajectory. Upon equilibration at 300 K, the rotated interlayer system provided a first visual representation of the formation of interlayer CBs between BTDZ molecules in the two adjacent organic layers (Figure 2b; Movie S1, with highlighted CB in Figure S3, Supporting Information). CB was also observed, albeit to a lesser extent, between BTDZ within the same organic layer (i.e., intralayer CB), further enhancing the structural stability (Figure S4, Movie S2, Supporting Information).

To validate finite temperature simulations, upon equilibration of all four systems at 300 K and 1 atm with AIMD, the average distance between inorganic slabs was calculated through the distance between the neighboring Pb planes. Independent of the starting configuration, the resulting first‐neighbor distance was 18.6 Å (Figure S5a, Supporting Information), which served as a point of comparison with the experimental data (see discussion below). Furthermore, the intermolecular distance between N and S atoms of neighboring thiadiazol rings was computed after averaging over a 10 ns‐long classical pre‐equilibrated MD trajectory at 300 K and 1 atm (Figure S5b, Supporting Information) and was found equal to 3.8 Å, comparing favorably with typical CB distances ranging from 3.5 to 4.3 Å.^[^ ^29^, ^35^ ^]^

By analyzing AIMD trajectories of the energetically most stable rotated interlayer system, we identified the prevalent relative orientation of neighboring BTDZ molecules forming CBs. To this end, four conformational angles were defined as the molecule planarity angle φ, the substituent rotation angle ξ_1_, the off‐plane angle ω, and the ring rotation angle θ (Figure 2c and Figure S6, Supporting Information). The first two angles, φ, and ξ_1_, described the molecular conformation of one single BTDZ molecule, whereas the remaining two angles, ω, and θ, quantified the relative orientation of two interacting BTDZ molecules when forming CBs. Based on the computed histograms of the four angles (Figure S7, Supporting Information), BTDZ molecules were found to be primarily planar, with the methylammonium substituent rotated by ≈90° off the ring plane, whereas the dominant CB interaction motif entailed two BTDZ molecules at an off‐plane angle of 45° and rotated by 20° with respect to each other (Figure 2, inset). This statistically prevalent CB interaction pattern compares well with the most energetically favorable rotated interlayer conformation. Among the three different interaction patterns between neighboring BTDZ molecules (Figure S2, Supporting Information), we identified that, for the energetically most favorable rotated interlayer configuration, N_1_SN_1_S and N_2_SN_2_S CB motifs were equiprobable with occurrence probabilities of ≈38% each, whereas the N_1_SN_2_S CB motif appeared less probable with an occurrence probability of 24%. This can be ascribed to the fact that, for an N_1_SN_2_S CB to form, the methylammonium substituents must lie on the same side of the interacting BTDZ molecules, a condition which is not easily satisfied in an RP perovskite, suggesting that multiple configurations may co‐exist in the materials, which was analyzed experimentally.

### 2.2 S‐Mediated Low‐Dimensional Hybrid Perovskite Materials

Having established the capacity of the BTDZ to form layered hybrid perovskites through CB theoretically, the corresponding thin films were experimentally analyzed. The spacer was synthesized by the protonation of the BTDZ amine precursor, as detailed in the Experimental Section and the Supporting Information (Figures S9,S10, Supporting Information). Subsequently, the spacer precursor, (BTDZ)I, was used to prepare materials through the reaction with stoichiometric amounts of PbI_2_ (for *n* = 1) and FAI (*n* > 1 compositions) by solution‐processing (spin‐coating) of films onto appropriate substrates and mechanosynthesis of powders by ball milling, followed by annealing at 150 °C. While the focus has been on the iodide‐based compositions, which are assumed to be more relevant in photovoltaics, we also analyzed bromide analogs based on (BTDZ)Br, for comparison.

**Figure 3:**
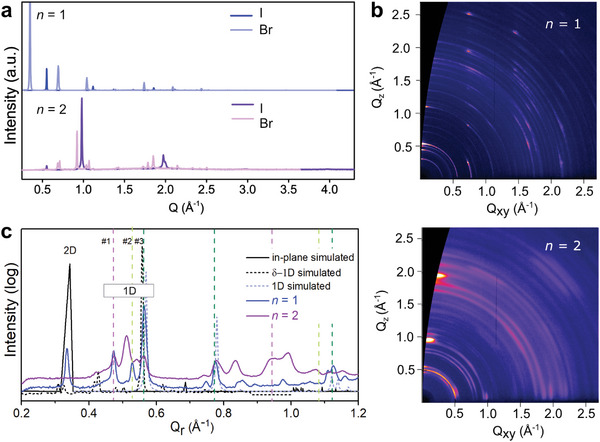
Structural characteristics of LD hybrid S‐mediated perovskites. a) XRD patterns of (BTDZ)X‐based LD perovskite thin films based on *n* = 1 (blue) and *n* = 2 (purple) nominal compositions (X = I, Br). b) GIWAXS reciprocal space maps of thin films based on *n* = 1 (top) and *n* = 2 (bottom) (BTDZ)_2_FA_n‐1_Pb_n_I_3n+1_ nominal composition on glass and c) the corresponding XRR scans probing the structure in comparison to simulated powder data of the 2D phase featuring CB (black), face‐sharing 1D (based on the X‐ray crystal structure data in dashed grey), and edge‐sharing 1D‐δ structures (dashed black). Bragg peaks of the additional LD polymorphs are highlighted in dashed lines (#1–#3, pink and green dashed lines). Further analysis of LD structures is detailed in Tables S2,S3, and Figures S11–S13, Supporting Information.

**Figure 4:**
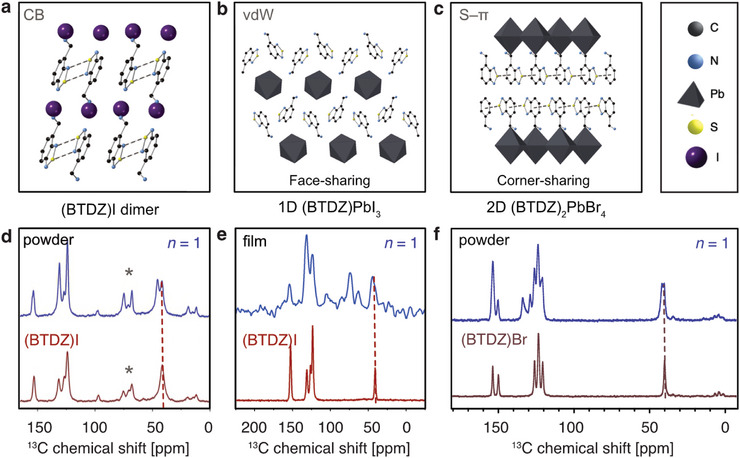
LD structures determined using single crystal X‐ray diffraction and NMR spectroscopy. a) (BTDZ)I (spacer; dashed lines show the CB interactions), b) 1D (BTDZ)PbI_3_ (face‐sharing octahedra stabilized by van der Waals (vdW) interactions visualized in Figure S12, Supporting Information), c) 2D (BTDZ)_2_PbBr_4_ (*n* = 1) (corner‐sharing octahedra; stabilized via S–π interactions indicated by dashed lines, with a complementary analysis in Figure S13, Supporting Information). The crystal structures (CCDC 2342700–2342702) and parameters are detailed in Table S4 (Supporting Information). d–f) ^13^C CP MAS NMR spectra of (BTDZ)X and (BTDZ)_2_PbX_4_ (*n* = 1) nominal stoichiometry (X = I is shown in panels d,e whereas X = Br in f). The thin films based on (BTDZ)_2_PbI_4_ (*n* = 1) nominal stoichiometry are shown in e). Asterisk indicates spinning side bands. Complementary ^13^C and ^14^N NMR spectra are shown in Figures S9,S10, and S14 (Supporting Information).

The structural properties of materials were studied by X‐ray diffraction (XRD) and solid‐state nuclear magnetic resonance (NMR) spectroscopy. XRD patterns of thin films (**Figure** **3**a) exhibited distinct reflections below *q* < 1 Å^−1^ for the nominal *n* = 1 compositions, which can be associated with a low‐dimensional (LD) phase that is preferentially oriented parallel to the substrate.^[^ ^17^, ^18^, ^19^ ^]^ In contrast, XRD patterns of *n* > 1 compositions suggested mixed orientations by changes in relative Bragg peak intensities.^[^ ^19^, ^20^ ^]^ This is in accordance with the observations by grazing‐incidence wide‐angle X‐ray scattering (GIWAXS) measurements, revealing the presence of several Bragg peaks with *q* < 1 Å^−1^ that can be attributed to LD structures (Figure 3b; Figure S11, Supporting Information).^[^ ^11^ ^]^ The *n* = 1 composition exhibited a mixture of LD structures revealed by diffraction peaks below *q* = 1 Å^−1^, which were assumed to be associated with co‐existing 2D and 1D phases (labeled 2D, 1D, and #1–3 in Figure 3c; Figure S11, Supporting Information, with detailed assignment in Tables S2,S3, Supporting Information). In contrast to the *n* = 1, nominal *n* = 2 compositions display a reduced degree of orientational order with other co‐existing phases (Figure 3c; Figure S11, Supporting Information). This included the *n* = 1 composition structures and 3D FAPbI_3_ perovskite, without the typically observed hexagonal (δ) phase, suggesting a contribution to the stabilization of the 3D α‐FAPbI_3_ perovskite phase. The X‐ray reflectivity (XRR) scans measuring the vertical stacking distance corroborated the existence of multiple phases (Figure 3c; Figure S11, Supporting Information), whereas the exact composition and contribution of LD phases were found to be substrate‐dependent (Figures S11,S12, Supporting Information). The scattering of films of *n* = 1 nominal composition on glass featured a structure with out‐of‐plane stacking of 18.8 Å, matching the radial distribution function of the most stable simulated 2D configuration featuring CB (18.6 Å, Figure S5, Supporting Information). The propensity to form such layered (2D) phases was affected by the counter ion, and the 2D structure was predominant for (BTDZ)_2_PbBr_4_ (Figure 3a; Figure S13, Supporting Information). The analysis of (BTDZ)_2_PbBr_4_ films on glass revealed two co‐existing 2D phases, which was confirmed by the GIWAXS analysis of films of *n* = 2 nominal composition (Figure S13, Supporting Information). One of the 2D structures was stabilized by S–π‐interactions (Figure 1d, bottom), as revealed by the corresponding X‐ray single crystal structure (**Figure** **4**c; Figure S13, Supporting Information). The origin of the co‐existing 2D phase was further examined theoretically to suggest a CB‐mediated structure, which was comparable to the I‐based RP system (Figure 2; Figure S13, Supporting Information), energetically close to the more stabilized S–π‐based 2D structure (Table S1, Supporting Information), forming a mixture of S‐mediated 2D structures.

Further analysis was performed to identify the nature of the remaining LD phases. The dominant LD structure (corresponding to the signals labeled as #3 with higher order reflections in Figure 3c) was found to be a 1D (BTDZ)PbI_3_ phase featuring a face‐sharing octahedral framework, as revealed by the corresponding X‐ray single crystal structure (Figure 4a,b; Figure S12, Supporting Information). The contributing LD polymorphs (indicated by signals labeled by #1 and #2 in Figure 3c) were found to correspond to an edge‐sharing 1D δ‐phase stabilized by interactions between BTDZ moieties (Figure S5, Supporting Information). The simulation of the 1D δ‐phase revealed a comparable XRD pattern to the experimental ones (Figure 3c; Figure S11, Supporting Information), indicating that such 1D δ‐phases could also form. These 1D phases were more apparent for the thin films based on *n*  = 2 nominal compositions (Figures S11,S12, and Tables S2,S3, Supporting Information). Moreover, some *n* = 2 compositions on glass also exhibited a reflection at 0.31 Å^−1^ that was likely to correspond to a 2D *n* = 2 RP phase, featuring a preferred orientation (Figure S11, Supporting Information). A comparable orientation was observed for the co‐existing 3D perovskite phase (Figure S11, Supporting Information), indicating a templating role of BTDZ‐based 2D phases. This overall suggests that BTDZ‐based perovskite materials are defined by a mixture of LD phases that are stabilized via S‐mediated interactions, determining the resulting properties.

To gain further insight into the atomic‐level interactions, we used ^1^H → ^13^C Magic Angle Spinning (MAS) solid‐state NMR spectroscopy of LD perovskites and thin films (Figure 4d–f; Figure S14, Supporting Information).^[^ ^36^, ^37^ ^] 13^C and ^15^N cross‐polarization (CP) MAS NMR spectra of the composition nominally corresponding to (BTDZ)_2_PbI_4_ (*n* = 1) accessed by mechanosynthesis and solution‐processing in films revealed a mixture of unreacted (BTDZ)I and a new material (Figure 4d,e), which we attribute to the 1D (BTDZ)PbI_3_ phase identified using single crystal X‐ray structure analysis (Figure 4b). Similarly, ^13^C CP MAS NMR spectrum of the nominally (BTDZ)_2_PbBr_4_ (*n* = 1) composition shows a mixture of unreacted (BTDZ)Br and a new phase (Figure 4f), which can be ascribed to the 1D (BTDZ)PbBr_3_. While mechanosynthesis uniquely leads to the formation of this LD polymorph, we could access the corresponding 2D (BTDZ)_2_PbBr_4_ phase (*n* = 1; Figure 4c; Figure S13, Supporting Information) using mild crystallization conditions. We also isolated a corresponding 1D structure from the same mother liquor, although the crystals were not high enough quality to refine the position of organic cations. This suggests that BTDZ‐based 1D structures are more thermodynamically stable than their 2D RP phases under experimental conditions. These hybrid BTDZ‐based LD materials were thereafter assessed for their optoelectronic characteristics which define their functionality.

**Figure 5:**
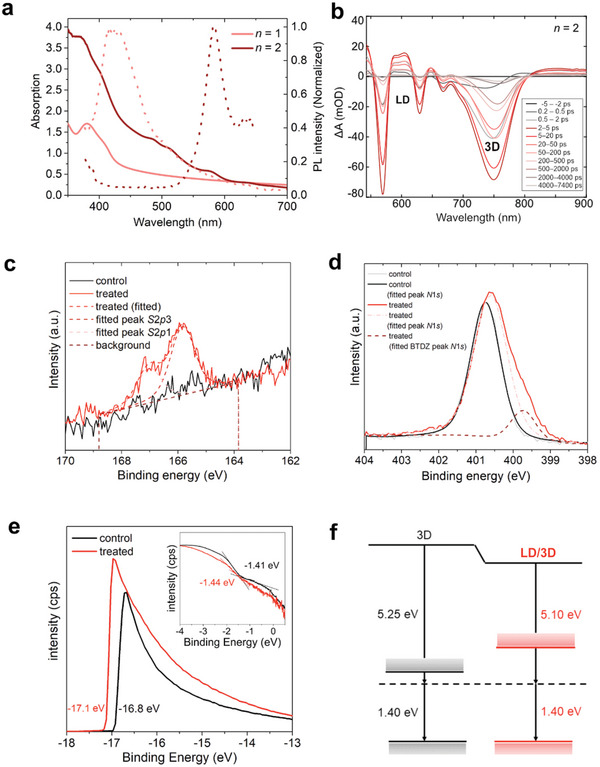
Structural and optoelectronic properties of LD and LD/3D perovskite thin films. a) UV‐vis absorption (full) and steady‐state PL (dashed) spectra of a film with composition nominally corresponding to (BTDZ)_2_PbI_4_ (*n* = 1, light red) and (BTDZ)_2_FAPb_2_I_7_ (*n* = 2, dark red). b) Transient absorption spectra of (BTDZ)_2_FAPb_2_I_7_ films at varying time delays upon excitation at 515 nm, indicating signals characteristic for co‐existing LD and 3D phases. c) S 2*p* and d) N 1*s* XPS core level spectra of 3D perovskite films without (control) and with (treated) (BDTZ)I layers. e) UPS spectra of the control and treated perovskite films, with the secondary electron cut‐off (SECO) for determining the work function. The binding energy of the valence band spectra is shown in the inset. f) Energy level diagram for 3D and LD/3D films.

The optical properties of thin films were investigated by steady‐state UV‐vis absorption and photoluminescence (PL) spectroscopy (**Figure** **5**a). The absorption spectra revealed excitonic peaks in the range of 350–400 nm, consistent with the formation of LD perovskite structures.^[^ ^38^, ^39^ ^]^ In the case of *n* = 2 nominal compositions, an excitonic peak was observed in the 500–600 nm range, accompanied by additional peaks, in accordance with the presence of mixed phases (Figure S15, Supporting Information). This was further reflected in the PL spectra, demonstrating emission at ≈425 nm for the *n* = 1 and 550–600 nm for the *n* > 2 compositions (Figure S15, Supporting Information), corroborating the mixture of LD (i.e., 1D and 2D) phases.^[^ ^38^, ^39^, ^40^ ^]^

The electronic interaction between the organic spacer and the inorganic slabs was further assessed by transient absorption (TA) spectroscopy and UV‐vis spectroelectrochemistry (SEC; Figure 5b; Figures S15,S16, Supporting Information). Upon excitation of the perovskite films of (BTDZ)_2_FAPb_2_I_7_ (*n* = 2) nominal composition at 515 nm, a distinct positive feature emerges ≈600 nm in the TAS spectra, possibly corresponding to a new species. This was accompanied by an additional bleaching signal between 550 and 600 nm associated with the presence of the LD perovskite phase, as well as 750 nm, in accordance with co‐existing 3D phase (Figure 5b; Figure S17, Supporting Information). The new species was not apparent across perovskite compositions, and it was further investigated by UV‐vis SEC and electron paramagnetic resonance (EPR) spectroscopy to assess the possibility of a charge transfer between the organic and inorganic layers. The SEC measurements suggested that the reduction at −1.5 V led to a quantitative increase in the UV absorption bands, whereas the reduction in the potential range of the reversible couple with *E*_1/2_ of −1.9 V induced a significant change in the absorption spectrum (Figure S16a, Supporting Information). The observed bands disappeared upon re‐oxidation in the back scan, and the original absorption at 310 nm reappeared, which continuously decreased in intensity upon oxidation in the range between −1.5 and −0.1 V (Figure S16a, Supporting Information). The spectral series recorded during the reversible redox transformation showed a direct conversion, as evidenced by the isosbestic points in the spectra. Therefore, we assign the observed conversion to the BDTZ/BDTZ^•–^ reduction.^[^ ^41^ ^]^ The nature of radical anion species was further evidenced by EPR SEC (Figure S16b, Supporting Information). The EPR spectra recorded during the reduction of the dissolved (BTDZ)I, however, exhibited no paramagnetic species until the working electrode potential reached the point of the reversible reduction. The potential was maintained at −2.1 V for 5 min to accumulate the paramagnetic species. Subsequently, a single relatively narrow line was observed with a *g*_iso_ of 2.0048 ± 0.0002 and Δ*B*_pp_ of 0.18 mT (Figure S16b, Supporting Information), and its intensity varied according to the expected BDTZ/BDTZ^•–^ process. The *g*_iso_ is compatible with an organic radical featuring significant spin density delocalization on a nitrogen atom. This analysis suggests that, under the experimental conditions, electron exchange is unlikely to occur spontaneously between the organic and the inorganic layers, which is in accordance with the TAS that does not unambiguously confirm charge transfer between the layers. The in‐plane conductivity of the (BTDZ)_2_PbI_4_ (*n* = 1) was thereby measured in lateral devices by depositing Au electrodes onto the perovskite films on glass. Electrical characterization of the lateral current revealed that the perovskite exhibits an average conductivity of 6.7 × 10^−4^ mS cm^−1^ (Figure S18, Supporting Information) that is significantly higher than values previously reported for 2D (*n* = 1) perovskite films, despite the mixture of LD phases.^[^ ^42^ ^]^ While vertical out‐of‐plane conductivities are more relevant for photovoltaics, LD perovskite networks can facilitate charge transfer^[^ ^43^ ^]^ and contribute to device performance,^[^ ^44^, ^45^, ^46^ ^]^ suggesting a beneficial impact of S‐mediated networks.

### 2.3 S‐Mediated Interactions in Hybrid Perovskite Photovoltaics

The effectiveness of BTDZ‐based layered hybrid perovskites in photovoltaics was analyzed in LD/3D perovskite heterostructures by incorporating organic spacer(s) onto 3D perovskites to form LD overlayers upon annealing in n‐i‐p perovskite solar cells. In addition, we analyzed p‐i‐n devices containing (BTDZ)I within their 3D perovskite structure. For this purpose, we focused on the conventional Cs_0.05_FA_0.90_MA_0.05_Pb(I_0.95_Br_0.05_)_3_ perovskite composition. The structural analysis of the resulting LD/3D perovskite films by XRD suggested that the presence of (BTDZ)I did not cause substantial changes in the crystal structure of the 3D perovskite (Figure S19a, Supporting Information). Similarly, the 3D perovskite structure was preserved when applying (BTDZ)I as an additive (Figure S19b, Supporting Information). Upon overlayer treatment, low‐angle reflections below 10° were observed, suggesting the formation of an LD phase, which was not apparent when using (BTDZ)I as an additive, presumably due to low concentration, since the use of a higher concentration of the (BTDZ)I revealed a signal below 10° associated with the LD structures (Figure S19, Supporting Information). Moreover, X‐ray photoemission spectroscopy (XPS) measurements confirmed the formation of the BTDZ overlayer. The untreated (control) 3D perovskite films exhibited no detectable Sulphur (S) signal, whereas a distinct S 2*p* doublet emerged at 165.8 eV consistent with the expected binding energy in a benzothiadiazole unit (Figure 5c).^[^ ^47^ ^]^ The N 1*s* spectrum of the treated sample also showed a noticeable contribution at a binding energy of 399.75 eV, consistent with benzothiadiazole (Figure 5d).^[^ ^47^ ^]^ The presence of BDTZ was also apparent in the C 1*s* spectrum, which in the treated sample exhibited an enhanced C─N species at a binding energy of 286.5 eV (Figure S20, Supporting Information). These measurements validate the presence of BTDZ on the surface, although Pb 4*f*, I 3*d*, and Cs 3*d* spectra remain unaffected (Figure S20, Supporting Information).^[^ ^48^, ^49^ ^]^ The formation of LD/3D heterostructures was confirmed by transmission electron microscopy (TEM), evidencing LD phases at the interface between the 3D perovskite and the hole‐transport material (Figure S21, Supporting Information). This interaction affected the morphology of the films, as evidenced by TEM and scanning electron microscopy (SEM), highlighting the differences in surface morphology between the control and BTDZ‐treated films (Figure S22, Supporting Information). However, this did not induce significant changes in the optical properties, as suggested by transient absorption and PL spectroscopy (Figures S23,S24, Supporting Information). The band alignment of the BTDZ‐treated layers was thereby examined by ultraviolet photoemission spectroscopy (UPS; Figure 5e). The work function showed a difference of 0.15 eV between reference and treated films, whereas the VB maxima remained unchanged (Figure 5f). Considering higher bandgap of LD phases, this suggests that BTDZ‐treated perovskite surface offers a suitable energy level alignment for hole extraction with effective blocking of electrons.^[^ ^50^ ^]^

**Figure 6:**
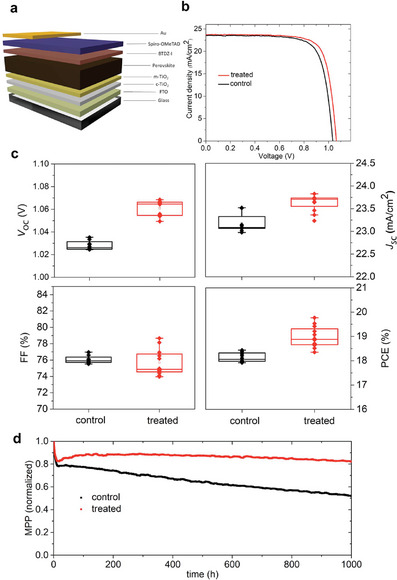
Photovoltaic performances in LD/3D perovskite solar cells. a) Schematic of the n‐i‐p device architecture. b) *J–V* curves of the champion control and BTDZ‐treated devices under the AM 1.5G illumination. c) Box charts illustrating the statistical distribution of open‐circuit voltage (*V*_OC_), short‐circuit current density (*J*_SC_), fill factor (FF), and power conversion efficiency (PCE) for control and BTDZ‐treated perovskite devices for 12 devices. d) Evolution of the PCE during operation by MPP tracking of control and BTDZ‐treated perovskite solar cells in N_2_ atmosphere under 1 sun irradiation at ambient temperature.

We thereby investigated the photovoltaic performance using devices that apply BTDZ at the interface with the hole‐transport layer in fluorine‐doped tin oxide (FTO)/compact‐TiO_2_/mesoporous‐TiO_2_/ Cs_0.05_FA_0.90_MA_0.05_Pb(I_0.95_Br_0.05_)_3_/ BTDZ/2,2′,7,7′‐tetrakis(*N*,*N*‐di‐4‐methoxyphenylamino)−9,9′‐spirobifluorene (Spiro‐OMeTAD)/Au n‐i‐p device configuration (**Figure** **6**a). The photovoltaic metrics were determined from the current–voltage (*J–V*) characteristics (Figure 6b). The treated devices exhibited an average increase in open‐circuit voltage (*V*_OC_) of 40 mV and an average increase in short‐circuit current (*J*_SC_) of 0.6 mA compared to the untreated ones, whereas the fill factor (FF) did not show a significant difference (Figure 6c). The incident photon‐to‐current efficiency (IPCE) spectra showed integrated short‐circuit currents that were comparable to those measured by the *J–V* characteristics (Figure S25, Supporting Information), excluding any significant spectral mismatch under the measurement conditions. Consequently, the devices demonstrated an enhanced power conversion efficiency (PCE) of up to 19.7% as compared to 18.6% for the control.

We compared these observations with the use of the spacer in p‐i‐n devices based on indium‐doped tin oxide (ITO)/NiO_x_/2‐(3,6‐dimethoxy‐9*H*‐carbazol‐9‐yl)ethyl]phosphonic acid (MeO‐2PACz)/ Cs_0.05_FA_0.90_MA_0.05_Pb(I_0.95_Br_0.05_)_3_:BTDZ/C_60_/bathocuproine (BCP)/Au architectures, suggesting comparable improvements in photovoltaic performance, with the average PCE increase from 17.4% to 18.3% (Figures S25,S26, Supporting Information), primarily due to the increase in the *V*_OC_. The performance could be further improved by applying (BTDZ)Br derivatives at the interface (Figure S27, Supporting Information). Time‐resolved photoluminescence (TRPL) measurements (Figure S28, Supporting Information) revealed a minor increase in the carrier lifetimes upon treatment (Table S5, Supporting Information), indicating contributions to the suppression of nonradiative recombination, in accordance with the improvements in the *V*_OC_, which was consistent with the TAS showing that the ground state bleach is longer lived for mixed‐dimensional heterojunctions (Figure S24, Supporting Information). The consistency of the effects across different device architectures also suggested the generality of the approach in terms of improvements in photovoltaic performances, which can be optimized in the future. This also applies to the quasi‐2D perovskite solar cells, which for *n* = 4 nominal compositions feature performances that are already comparable to some of the best‐performing FA‐based quasi‐2D perovskite solar cells reported to date (Figure S29, Supporting Information) with PCE up to 7.5%^[^ ^22^ ^]^ without further optimization, suggesting the potential of S‐mediated interactions in photovoltaics.

The impact of this supramolecular strategy is more relevant for the operational stability, which was analyzed by monitoring the evolution of the maximum power point (MPP) of n‐i‐p devices under continuous irradiation in a nitrogen atmosphere (Figure 6d). While the control devices exhibited a drop to 50% of their initial performance after 1000 h, the treated devices displayed improved stability, maintaining over 80% of their performance. The drop in performance during the first few hours was comparable to other halogen‐bonded perovskite solar cells,^[^ ^26^, ^27^ ^]^ although the overall stability surpasses them. This was likely associated with the increase in the hydrophobicity of the perovskite upon treatment, as evidenced by the contact angle measurements (Figure S30, Supporting Information), which is of interest to their resilience against moisture but also improving the contact with the hole‐transport layer^[^ ^25^, ^26^, ^27^ ^]^ that is relevant to their performance and operational stability. This opens new perspectives for S‐mediated interactions in perovskite photovoltaics.

## 3 Conclusion

In summary, we developed novel low‐dimensional (LD) hybrid perovskite materials by incorporating functionalized benzothiadiazole‐based (BTDZ) organic spacers that assemble through S‐mediated interactions, including chalcogen bonding (CB) and competing S–π interactions. The formation of the LD structures was probed by a combination of techniques, including X‐ray diffraction and solid‐state NMR spectroscopy, complemented by theoretical investigations, including molecular dynamics simulations and density functional theory calculations, revealing the structural complexity of LD phases stabilized by S‐mediated interactions. The effect on the optoelectronic properties was evaluated through UV‐vis absorption, photoluminescence, transient absorption spectroscopy, and UV‐vis/EPR Spectro‐electrochemistry to verify LD perovskite formation and assess electronic exchange between the layers, whereas conductivity and UPS measurements suggested favorable energetic alignment with the BTDZ‐overlayer‐containing perovskite in solar cells. We thereby applied an LD perovskite overlayer at the interface with the hole‐transport layers in conventional n‐i‐p and p‐i‐n perovskite solar cell architectures, demonstrating improvements in the performances and operational stabilities of the devices. While further optimization of thin film fabrication and device engineering could offer more competitive photovoltaic performances, this unprecedented approach provides a proof‐of‐concept for the utility of unorthodox S‐mediated interactions in the design of perovskite materials and photovoltaics. This sets the stage for a versatile supramolecular strategy in the advancement of LD perovskites and their application.

## Conflict of Interest

The authors declare no conflict of interest.

## Author Contributions

W.L., S.K., and N.L. contributed equally to this work. The project was conceptualized by J.V.M. and led by W.L., who conducted synthesis and characterization with the support of E.C. and P.A.G. under the supervision of J.V.M. Furthermore, W.L. fabricated and characterized thin films and n‐i‐p photovoltaic devices with the support of S.‐J.K, who fabricated and characterized p‐i‐n devices and performed complementary characterization, including TRPL, under the supervision of J.‐S.K. Throughout the study, N.L. performed theoretical investigations with the support of M.D., V.C., L.A., and V.S. under the supervision of U.R., whereas M.Z. performed SEC and EPR measurements. L.P., B.G., and D.J.K. conducted the solid‐state NMR spectroscopy whereas A.T. and Z.V.O. performed transient absorption measurements under the supervision of J.‐E.M. and S.F., respectively. L.M., E.K., and P.Z. performed GIWAXS measurements and analysis under the supervision of A.H. and F.S., W.B. isolated single crystals while M.S. carried out single crystal XRD and structure refinement. Finally, L.A.G.‐L. performed the lateral conductivity measurements and M.D. performed the XPS and UPS measurements under the supervision of Y.V. All authors contributed to the manuscript.

## Supporting information

Supporting Information

Supplemental Movie 1

Supplemental Movie 2

## Data Availability

Data presented here can be accessed at the following DOI:10.5281/zenodo.12668415, and it is available under the license CC‐BY‐4.0 (Creative Commons Attribution‐ShareAlike 4.0 International).
