# Supplementary material for: From Chalcogen Bonding to S–π Interactions in Hybrid Perovskite Photovoltaics
Source: Adv Sci (Weinh). 2024 Jul 3;11(32):2405622. doi: 10.1002/advs.202405622 (PMC11347993; doi:10.1002/advs.202405622)
Supplement: Supplementary file 1 — Supporting Information [file ADVS-11-2405622-s003.pdf]

ADVANCED SCIENCE
Open Access

Supporting Information

for *Adv. Sci.*, DOI 10.1002/advs.202405622

From Chalcogen Bonding to S–π Interactions in Hybrid Perovskite Photovoltaics

Weifan Luo, SunJu Kim, Nikolaos Lempesis, Lena Merten, Ekaterina Kneschaurek, Mathias Dankl, Virginia Carnevali, Lorenzo Agosta, Vladislav Slama, Zachary VanOrman, Miłosz Siczek, Wojciech Bury, Benjamin Gallant, Dominik J. Kubicki, Michal Zalibera, Laura Piveteau, Marielle Deconinck, L. Andrés Guerrero-León, Aaron T. Frei, Patricia A. Gaina, Eva Carteau, Paul Zimmermann, Alexander Hinderhofer, Frank Schreiber, Jacques-E. Moser, Yana Vaynzof, Sascha Feldmann, Ji-Youn Seo, Ursula Rothlisberger* and Jovana V. Milić**

Supporting Information

From Chalcogen Bonding to S-π Interactions in Hybrid Perovskite Photovoltaics

Weifan Luo[1#], SunJu Kim[2#], Nikolaos Lempesis[3#], Lena Merten[4], Ekaterina Kneschaurek[4], Mathias Dankl[3], Virginia Carnevali[3], Lorenzo Agosta[3], Vladislav Slama[3], Zachary VanOrman[5], Milosz Siczek[6], Wojciech Bury[6], Benjamin Gallant[7], Dominik J. Kubicki[7], Michal Zalibera[8], Laura Piveteau[9], Marielle Deconinck[10,11], L. Andrés Guerrero-León[10,11], Aaron T. Frei[12], Patricia A.Gaina[1], Eva Carteau[1], Paul Zimmermann[4], Alexander Hinderhofer[4], Frank Schreiber[4], Jacques-E. Moser[12], Yana Vaynzof[10,11], Sascha Feldmann[5], Ji-Youn Seo[2*], Ursula Rothlisberger[3*], and Jovana V. Milić [1,3*]

[1]Adolphe Merkle Institute, University of Fribourg, 1700 Fribourg, Switzerland; [2]Department of Nanoenergy Engineering, Pusan National University, 46241 Busan, South Korea; [3]Laboratory of Computational Chemistry and Biochemistry, Institute of Chemical Sciences and Engineering, École Polytechnique Fédérale de Lausanne, 1015 Lausanne, Switzerland; [4]Institute of Applied Physics, University of Tübingen, 72076 Tübingen, Germany; [5]Rowland Institute, Harvard University, Cambridge MA 02142, USA; [6]Faculty of Chemistry, University of Wrocław, 50-383 Wrocław, Poland; [7]School of Chemistry, University of Birmingham, B15 2TT Birmingham, UK; [8]Institute of Physical Chemistry and Chemical Physics, Slovak University of Technology, 81237 Bratislava, Slovakia; [9]Laboratory of Magnetic Resonance, EPFL, 1015 Lausanne, Switzerland; [10]Chair for Emerging Electronic Technologies, Technical University of Dresden, 02062 Dresden, Germany; [11]Leibniz Institute for Solid State and Materials Research Dresden, Germany; [12]Photochemical Dynamic Group, Institute of Chemical Sciences and Engineering, École Polytechnique Fédérale de Lausanne, 1015 Lausanne, Switzerland; [#]These authors contributed equally to this work. [*]Corresponding authors: j-y.seo@pusan.ac.kr; ursula.roethlisberger@epfl.ch; jovana.milic@unifr.ch.

Table of Contents

Experimental Section 2

Supporting Data 8

Supplementary Discussion 29

Reference 30

Experimental Section

Materials and Methods

Commercial materials: Lead iodide (PbI_2, 99.99%) and cesium iodide (CsI, 99.99%) were purchased from Tokyo Chemical Industry Co., Ltd. Lead bromide ($PbBr_2$, 99.99%), formamidinium iodide (FAI, 99.8%), and methylammonium bromide (MABr, 99.8%) were purchased from Greatcell Solar Materials Pty Ltd. Spiro-OMeTAD (>99.8%) was purchased from Luminescence Technology Corp. Anhydrous *N*,*N*-dimethylformamide (DMF), chlorobenzene (CB) and dimethyl sulfoxide (DMSO) were purchased from Sigma-Aldrich . Benzo[*c*][1,2,5]thiadiazol-4-ylmethanamine precursor was purchased from abcr GmbH.

Benzo[c][1,2,5]thiadiazol-4-ylmethanammonium iodide ((BTDZ)I) was synthesized by protonation of benzo[*c*][1,2,5]thiadiazol-4-ylmethanamine. HI (0.63 mL, 4.84 mmol, 57%) was added to a suspension of the precursor (0.5 g, 3.03 mmol) in ethanol (10 mL) at 0 °C. The mixture was stirred for 12 h, and the resulting solution was concentrated, resuspended in diethyl ether (10 mL), filtered, extensively washed with diethyl ether, and dried to yield (BTDZ)I (792.1 mg, 89.3%) as a pale-yellow powder. ^{1}H NMR (400 MHz, $(CD_3)_2SO$): δ 4.52 (2H, s), 7.78–7.86 (2H), 7.83 (*t*, *J* = 7.9 Hz), 7.85 (dd, *J* = 7.9, 2.0 Hz), 8.13 (1H, dd, *J* = 8.0, 2.0 Hz), 8.37 (3H, s) ppm. ^{13}C NMR (400 MHz, $(CD_3)_2SO$): δ 39.14, 122.07, 127.26, 129.56, 130.3, 153.52, 154.71 ppm. HRMS (ESI/QTOF) m/z: [M]+ calcd. for $C_7H_8N_3S^+$ 166.0433; found 166.0442.

Benzo[c][1,2,5]thiadiazol-4-ylmethanaminium bromide ((BTDZ)Br) was synthesized by protonation of benzo[*c*][1,2,5]thiadiazol-4-ylmethanamine. HBr (0.33 mL, 2.91 mmol, 48%) was added to a suspension of the precursor (0.3 g, 1.82 mmol) in ethanol (10 mL) at 0 °C. The mixture was stirred for 12 h and the resulting solution was concentrated, resuspended in diethyl ether (10 mL), filtered, washed with diethyl ether and isopropanol, and dried under vacuum to yield (BTDZ)Br (260 mg, 58%) as a pale-yellow powder. ^{1}H NMR (400 MHz, $(CD_3)_2SO$): δ = 8.41 (bs, 3H), 8.15 (m, 1H), 7.82 (m, 1H), 4.57 (s, 2H) ppm; ^{13}C NMR (101 MHz, $(CD_3)_2SO$): δ = 154.71, 153.53, 130.28, 129.55, 127.28, 122.05, 39.09 ppm.

Perovskite powders were prepared using mechanosynthesis based on $(BTDZ)_2PbX_4$ (n =1; X = I, Br) nominal compositions. Stoichiometric amounts of PbX_2 and (BTDZ)X were ground with steel beads in a Retsch Ball Mill MM-200 using a 10 mL grinding jar and a ⌀10 mm ball. The grinding process was carried out for 30 min at 25 Hz, followed by subsequent annealing at 150 °C for 15 min to obtain the final powder.

Perovskite thin films of $(BTDZ)_2PbX_4$ (n = 1) or $(BTDZ)_2FAPb_2X_7$ (n = 2) nominal compositions were prepared using solution-processing of stoichiometric amounts of (BTDZ)X, PbX_2, and FAX in concentrations of 0.4 M (X = I, Br). The solutions were mixed to obtain the perovskite precursor solution, which was applied onto the prepared glass substrate using a two-step spin-coating technique. In the initial phase, the coating process was executed at a spin speed of 1000 rpm, with an acceleration rate of 200 rpm/s,

sustained for 10 s. Subsequently, the subsequent step was performed at 6000 rpm, with an acceleration rate of 2000 rpm/s, for a period of 30 s. The substrate was subsequently annealed at 150 °C for 15 min.

Direct (n-i-p) solar cell fabrication involved FTO (fluorine-doped tin oxide) glass substrates that were sequentially cleaned with 2% Hellmanex solution, isopropanol, and ethanol in an ultrasonic bath for 15 min and then dried with N_2. The cleaned FTO glass substrates were treated with UV-Ozone for 5 min before the deposition of TiO_2. The TiO_2 layer was deposited by aerosol spray pyrolysis using oxygen as a carrier gas. For approximately 30 samples of 1.5 x 2.5 cm (112.5 cm^2), 0.2 mL of acetylacetone and 0.3 ml of titanium diisopropoxide bis(acetylacetonate) stock solution (75 wt. % in isopropanol) is diluted in 4.5 mL of ethanol for a total of 5 ml of solution (10% conc.). Substrates are heated to 450 °C for 15 min and 30 min after the spray of the precursor solution. A layer of mesoporous TiO_2 (m-TiO_2) was deposited onto the compact TiO_2 (c-TiO_2) substrate through a spin-coating process lasting 10 se, conducted at 4000 rpm with an acceleration rate of 1000 rpm/s. This deposition was achieved utilising the 30NRD TiO_2 paste, with a quantity of 0.3 g per 2 mL of ethanol. Following this, the substrates dried at 100 °C, after which they underwent sintering at 450 °C for a duration of 30 min to form FTO/c-TiO_2/m-TiO_2. Subsequently, this substrate was coated with a perovskite solution that was prepared according to the method described below. For the hole-transport material, 100mg Spiro-OMeTAD was dissolved in 1.08 mL CB, doped by 23 µL Li-TFSI (1.8 mol/L in acetonitrile) and 39 µL tBP. The mixed Spiro-OMeTAD solution was spin-coated on the surface of the perovskite at 4000 rpm for 20 s with an acceleration rate of 2000 rpm/s. Finally, an 80 nm Au electrode was deposited by thermal evaporation through a shadow mask to form a device with an active area of 0.1 cm^2.

LD/3D perovskite layer was prepared by dissolving a mixture of PbI_2 (543.0 mg), $PbBr_2$ (21.0 mg), FAI (189.3 mg), MABr (6.4 mg), and CsI (15.8 mg) $Cs_{0.05}FA_{0.9}MA_{0.05}Pb(I_{0.95}Br_{0.05})_3$ with 5% excess of PbI_2 in 1 mL of DMF and DMSO mixture (4:1 v/v) at 70 °C. The perovskite active layer was deposited using an antisolvent method with chlorobenzene. The perovskite precursor solution was deposited on the freshly prepared FTO/c-TiO_2/m-TiO_2 substrate, and a two-step spin-coating method was applied. The first step was carried out at 2000 rpm with an acceleration rate of 200 rpm/s for 10 s. The second step followed at 6000 rpm with an acceleration rate of 2000 rpm/s for 30 s. CB (200 µL) was slowly dripped at 15 s before the second step. After this, the substrate was annealed at 150 °C for 10 min. To treat the device, we applied a 1 mg/mL solution of (BTDZ)X (X = I, Br) in ethanol, which was then spin-coated on the surface of the perovskite at 4000 rpm for 20 s with an acceleration rate of 2000 rpm/s followed by annealing at 130 °C for 5 min.

The inverted (p-i-n) planar perovskite solar cells were fabricated in a nitrogen-filled glovebox. First, the NiO-sputtered ITO glass substrate was cleaned by ultrasonic cleaning in isopropanol and ethanol and subsequently dried in oven. The ITO/NiO substrate was then heated at 300 °C for 10 min. The hole transport layer was deposited on the ITO/NiO substrate by spin-coating the MeO-2PACz ([2-(3,6-dimethoxy-9*H*-carbazol-9-yl)ethyl]phosphonic acid) solution at 3000 rpm for 30 s, followed by annealing at 100 °C for

10 min. The perovskite precursor solution was then spin-coated on the substrate in a one-step method at 3000 rpm for 10 s. The substrate was immediately put into a sample chamber connected to a vacuum pump, and the perovskite film was immediately exposed to low pressure for 15 s. The substrates were then transferred to a hot plate and annealed at 100 °C for 20 min. For the ETL, C_{60} was deposited using a thermal evaporator at a thickness of about 15 nm. The BCP solution was then spin-coated onto the C_{60} films at 4000 rpm and annealed at 70 °C for 5 min. Finally, a 100 nm Ag electrode was deposited by a thermal evaporator.

Ab initio calculations were performed within the Density Function Theory (DFT) framework as implemented in the CP2K software.[1] Both static optimisation and molecular dynamics simulations adopted the GGA-PBE functional with D3 dispersion corrections.[2] GTH pseudopotentials[3,4] together with the DZVP basis set[5] were utilised with an energy cutoff at 370 Ry. All three simulated n=1 RP systems (parallel in-plane, T-shaped interlayer and T-shaped intralayer) comprised a 4×4×2 slab of $(BTDZ)_2PbX_4$ with dimensions 27.67×27.67×40.5Å containing 32-unit cells for a total of 1376 atoms. Each system was then progressively heated up from 100 K to 300 K with a temperature step of 100 K by using the Bussi thermostat[6] with a 0.5 fs integration step. At each temperature increase, the systems were equilibrated for 8 ps. At 300 K, the systems were sampled for a total of 14 ps, the last 4 ps of which were averaged to compute the energy. The final equilibrated configurations were then used for the visualisation of CBs with the help of NCIPLOT,[7] and subsequently, the entire MD trajectory was analysed with in-house codes for the extraction of the conformational statistics (Fig. S7).

Classical MD simulations were performed with the LAMMPS software package.[8] The equations of motion were integrated using the Velocity-Verlet[9] algorithm with a 1 fs time step. Isothermal and isobaric conditions were maintained with the help of the Nosé-Hoover[10-11] thermostat and barostat with time constants 0.1ps and 0.35ps, respectively. Periodic boundary conditions were applied in all three directions. All classical systems, comprising a 9×9×2 slab of $(BTDZ)_2PbX_4$ with dimensions 62.26×62.26×40.5Å and a total of 6966 atoms, were equilibrated for 1ns at 300 K in the canonical NVT ensemble, where the total number of atoms N, the system's volume V and the temperature T were held constant. Next, production simulations were performed for 10 ns in the isothermal-isobaric NPT ensemble, where the sampling process took place every 500 fs for the computation of ensemble averages. Interatomic interactions for the perovskite part were described by an in-house developed and thoroughly validated classical force field.[12] The BTDZ cations were parametrised in the context of the OPLS all-atom force field by using LigParGen.[13] The van der Waals and electrostatic non-bonded interactions were calculated using a real-space cutoff radius of 10 and 8 Å, respectively, whereas the particle-particle-particle-mesh (PPPM) scheme[14] with an accuracy of 0.0001 (kcal/mol)/Å was used for the consideration of long-range electrostatic interactions.

The construction of the low-dimensional 1D δ-phase was realised starting from the δ-$FAPbI_3$ and δ-$CsPbI_3$ structures and expanding affinely the lattice vectors by 100% so that the BTDZ molecules could fit and

replace all FA and Cs, respectively. The built systems comprised a 4×3×1 supercell. Next, the systems were equilibrated with AIMD simulations at 300 K and 1 atm in the NPT ensemble. To consider relaxation modes with longer characteristic times, the equilibrated structures were replicated in all three dimensions 3×4×7 times for the initialisation of classical MD simulations for 20 ns in the NPT statistical ensemble. The XRD diffraction patterns of the equilibrated structures were generated using CrystalDiffract (CrystalMaker Software Ltd, Oxford, England; www.crystalmaker.com). Interestingly, the simulated XRD pattern of the 1D δ-phase originating from $CsPbI_3$ reproduced well the peaks of the experimental XRD diffraction patterns originally assigned to different layered polymorphs (Fig. S5 and Fig. S11). The organic component of this LD phase formed a continuous cross-linked network of BTDZ molecules interacting through CBs (Fig. S5).

***The relative formation energies (E_f)* were computed** for all considered systems with the help of AIMD simulations complemented by DFT calculations. Initially, each system was relaxed with AIMD simulations at 300 K and 1 atm. Following that, each equilibrated system was quenched down to 0 K. For the formation energies, we computed via DFT the energetic contribution of each ion of the system – Pb^{2+}, I^-, $BTDZ^+$, Br^- – as if it was isolated and we applied the following formula:

$$E_f = (E_s - \sum_i n_i E_i) / m \qquad \text{(Eq. 1)}$$

where E_s is the energy of the system, i is the ion type, n_i indicates the number of ions of type i in the system, E_i is the DFT energy of ion i and m is the number of stoichiometric units of the model system. This analysis identified the 2D $(BTDZ)_2PbBr_4$ system as the energetically most favourable one, whereas the LD systems were the least stable ones. The resulting energy relations are shown in Table S1. Similarly, the calculated projected density of states (PDOS) of all systems (Fig. S8) suggests that only the 2D $(BTDZ)_2PbI_4$ and 1D δ-phase systems contributed favourably towards supporting charge transfer, with the molecular orbitals of BTDZ of these systems overlapping with the bottom of the perovskite conduction band, thereby reducing the band gap. The remaining LD systems, i.e., 1D $(BTDZ)PbI_3$ and 2D $(BTDZ)_2PbBr_4$ (Fig. S8b,c) exhibited trapped states within the band gap, inhibiting potential charge transfer (Fig. S8a,d).

The solid-state nuclear magnetic resonance (NMR) spectra were recorded at 11.7 T (125 MHz for ^{13}C) and 20 T (213.79 MHz for ^{13}C, 86.15 MHz for ^{15}N). The 11.7 T instrument (EPFL) was equipped with a Bruker Avance III console and used 3.2 mm three-channel low-temperature magic angle spinning (MAS) probe. The samples were packed into 3.2 mm zirconia rotors under ambient conditions. The 20 T instrument (University of Warwick) was equipped with a Bruker Avance NEO console, and it used a 4 mm three-channel MAS probe. The samples were packed into zirconia rotors under ambient conditions.

X-ray diffraction measurements were conducted using a PANalytical Empyrean Series 2 instrument in Bragg-Brentano configuration. The instrument utilised Cu Kα radiation with a voltage of 40 kV and a current of 40 mA. For the specific purpose of grazing incidence measurements, the X-ray incidence angle

was set at 2°. Diffracted X-rays were detected during the experiments employing a PIXcel3D detector. The chemical composition of films was measured using *XPS* (AXIS SUPRA) with an Al Ka radiation source, and all binding energies were calibrated by C 1s (248.8 eV) as a reference.

Grazing incidence wide-angle X-ray scattering (GIWAXS) was employed to analyse the thin films deposited on microscope and ITO glass substrates. GIWAXS and XRR measurements were performed at DESY beamline P08.[15] The beam energy was 18 keV, the incidence angle of 0–0.3°, and the beam size was 300x100 µm. A Perkin Elmer XRD 1621 detector with a spatial resolution of 200 µm was used to record two-dimensional diffraction patterns with a sample-to-detector-distance of 700 mm. For the recording of XRR data, a Dectris Pilatus 100 K detector was used. Additional GIWAXS measurements were performed at the ESRF beamline ID10. An Eiger 4M detector was used for the diffraction measurement with a sample-to-detector distance of 625 mm. The beam energy was 22.5 keV with a beam size of 17 x 28.5 µm and an angle of incidence of 0-0.3°. All measurements were done in an inert nitrogen atmosphere.

Single crystal X-Ray diffraction data for (BTDZ)I, (BTDZ)PbI_3 and $(BTDZ)_2PbBr_4$ were collected on a Rigaku Oxford Diffraction XtaLAB Synergy-R DW diffractometer equipped with a HyPix ARC 150° Hybrid Photon Counting (HPC) detector at 100 K using Cu K_α (λ = 1.54184 Å). Data were processed using the *CrysAlisPro* software.[16] The structures were solved by intrinsic phasing with SHELXT[17] and refined by full-matrix least-squares methods based F^2 using SHELXL.[18] *The single crystals* were obtained by using the following procedures. *For (BTDZ)I*: The crystallisation process involved a slow crystallisation from a saturated solution of BTDZI in tetrahydrofuran. The structure is available on the Cambridge Structural Database under CCDC number 2342700. *(BTDZ)PbI_3:* In a 10 mL vial 5.9 mg (12.8 µmol) of PbI_2 was mixed with 7.5 mg (25.6 µmol) of BTDZI in 5 mL of tetrahydrofuran and sonicated until a clear yellow-orange solution was obtained. The vial was open and placed on a hotplate set to 85 °C. After evaporation of ca. 80% of the solvent volume, small crystals formed on the walls of the vial. The hot plate was turned off, the solution was allowed to cool down slowly to room temperature, and the colourless block crystals were collected. The structure of (BTDZ)PbI_3 is available on the Cambridge Structural Database under CCDC number 2342701. *$(BTDZ)_2PbBr_4$*: In a 20 mL vial 6.0 mg (16.3 µmol) of $PbBr_2$ was mixed with 8.0 mg (32.5 µmol) of BTDZBr in 10 mL of acetone and sonicated until a clear yellow-orange solution was obtained. The vial was opened and placed on a hotplate set to 65°C. After evaporation of ca. 90% of the solvent, small crystals formed on the walls of the vial. The hot plate was turned off, the solution was allowed to cool slowly to room temperature, and the colourless block crystals were collected. The structure of $(BTDZ)_2PbBr_4$ is available on the Cambridge Structural Database under CCDC number 2342702.

UV-vis absorption measurements were conducted using a Shimadzu UV-2600 spectrophotometer. ***Transient absorption spectra*** of perovskite films were recorded using a previously described setup[19] using modules supplied by Light Conversion, with a 1030 nm seed laser (PHAROS, Light Conversion, Yb:KGW lasing medium, 400 µJ pulse energy, 200 fs duration, 50 kHz repetition rate). The 515 nm or 343 nm pump

beam (850 μm in diameter) was generated from the seed using a harmonic generation unit (HIRO, Light Conversion) *via* non-linear crystals (BBO, lithium triborate). The probe beam (200 μm in diameter) was generated from the seed laser using supercontinuum generation in a sapphire crystal. The pump-probe delay was controlled over a range of 8 ns by changing probe path length via a multipass delay stage, and the pump was passed through an optical chopper (100 Hz), where a beamsplitter/photodiode combination was used to divide and sort measurements. The probe beam was passed into a grating spectrograph (Andor Kymera 193i, grating blaze wavelength 800 nm) and recorded using a Si NMOS photodiode array detector (256 pixels). TA experiments used a 515 or 343 nm pump at variable fluence (6.9 or 62 $\mu J/cm^2$) and a repetition rate of 50 kHz. The probe generated from the 1030 nm fundamental spanned from ~540 - 960 nm.

Steady-state photoluminescence (PL) spectra were acquired using a Fluorolog 322 spectrometer (Horiba Jobin Yvon iHr320) equipped with a CCD detector, using a slit width of 5 nm, and the excitation by a Xenon lamp at a wavelength of 350 nm. The band slit width during the measurements was set to 5 nm.

Time-resolved photoluminescence (PL) spectra were recorded using an excitation wavelength of 460 nm by an Edinburgh FS5 Spectrofluorometer.

EQE spectra were measured using an Oriel IQE-EQE 200B.

The cyclic voltammetry (CV) and spectroelectrochemistry (SEC) were performed in an acetonitrile solution with 0.2 M tetrabutylammonium hexafluorophosphate (nBu_4NPF_6) as a supporting electrolyte. The sample was washed in the nitrogen-filled glovebox in the electrolyte solution for 15 min, and the resulting solution was investigated by CV, UV-vis-SEC (in the glovebox), or EPR-SEC (in a sealed electrochemical cell). All experiments were done in a three-electrode arrangement with Pt auxiliary electrode (AE) and Ag wire as a pseudo-reference electrode (RE). Glassy carbon disk (1 mm diameter) was the working electrode (WE) in CV measurements, UV-vis-SEC used a honeycomb Pt WE, and the EPR-SEC experiment employed the Pt mesh as WE. The HEKA PG390 (Heka, Germany) potentiostat was used for potential control. All potential values reported herein are referenced vs. ferrocenium/ferrocene (Fc^+/Fc) redox couple. *EPR and UV-vis spectra* were recorded in situ during the reduction, using the X-band (9.5 GHz) EPR spectrometer PS-100 (Adani, Belarus) and Avantes optical spectrometer (Model AvaSpec-2048x14-USB2), respectively

XPS and UPS measurements were performed in an ultra-high vacuum chamber (ESCALAB 250Xi, base pressure of 2×10^{-10} mbar). XPS was measured using X-ray beam generated by XR6 monochromated Al Kα source (1486.6 eV) with a pass energy of 20 eV. UPS was performed using a double differentially pumped He gas discharge lamp emitting He I radiation (21.22 eV) with a pass energy of 2 eV and a bias of −5 V. *UPS analysis* was performed by plotting the spectra in log scale, in agreement with previous reports.[20]

Supporting Data

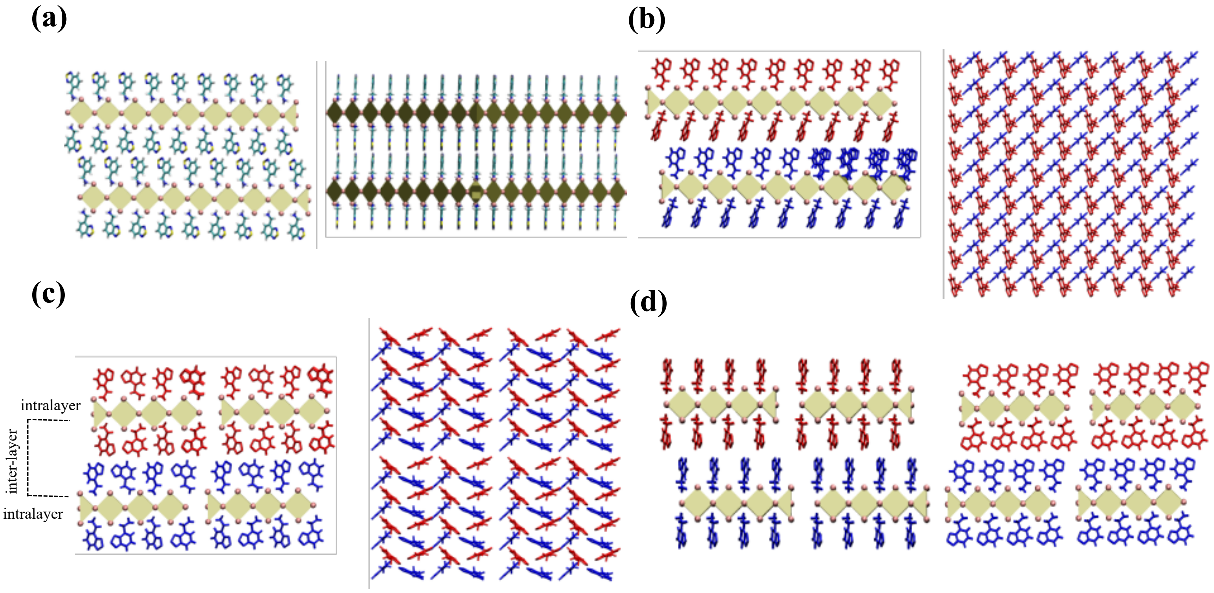

Fig. S1. The initial configuration used in CMD and AIMD simulations entailing the a) *parallel in-plane*, b) the *rotated interlayer*, c) the *rotated intralayer* and d) the *parallel displaced* conformations. In each part, two different perspectives are shown. In parts b), c) and d), the two organic layers are coloured differently (lower layer: blue, top layer: red). In parts b) and c), the inorganic layer is hidden in the top views for clarity.

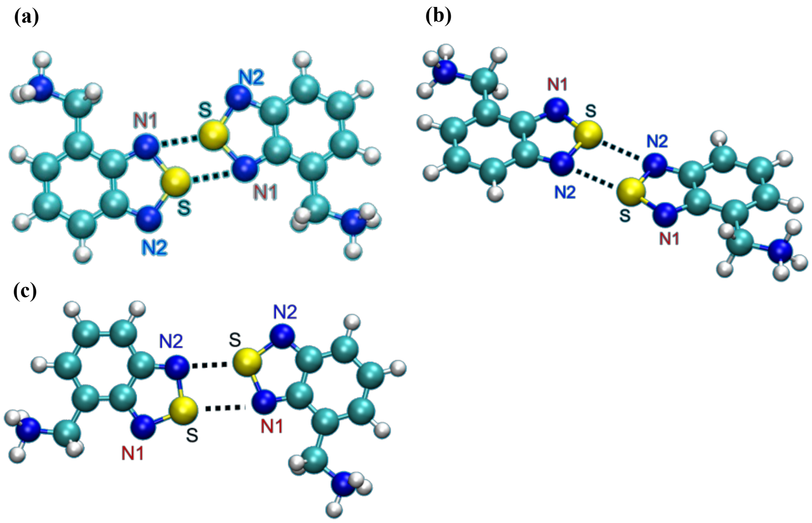

Fig. S2. Schematic of three different interaction patterns allowing CB formation. N_1 is the nitrogen atom closest to the methylammonium substituent, whereas CBs are depicted with black broken lines.

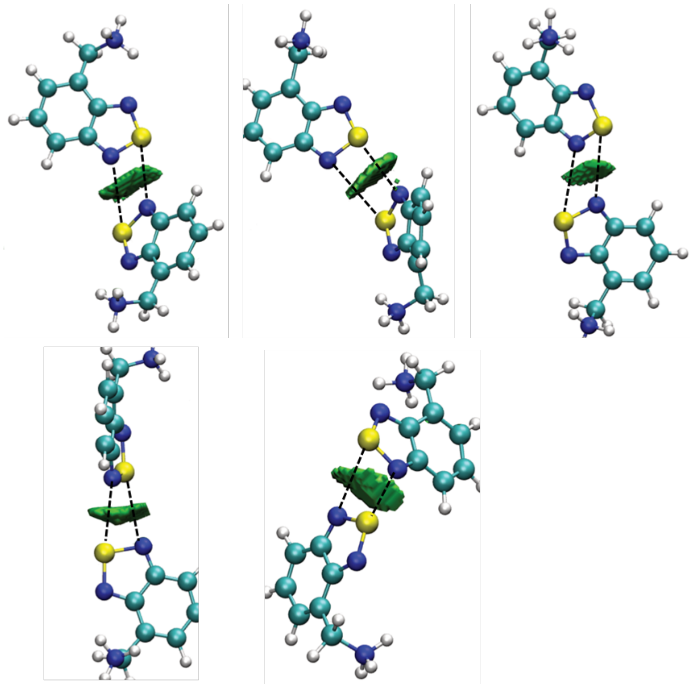

Fig. S3. Examples of interlayer CB formation from an equilibrated AIMD trajectory of the *rotated interlayer* model. In all cases, CBs are denoted with black broken lines and visualized via NCIPLOT green isosurfaces.

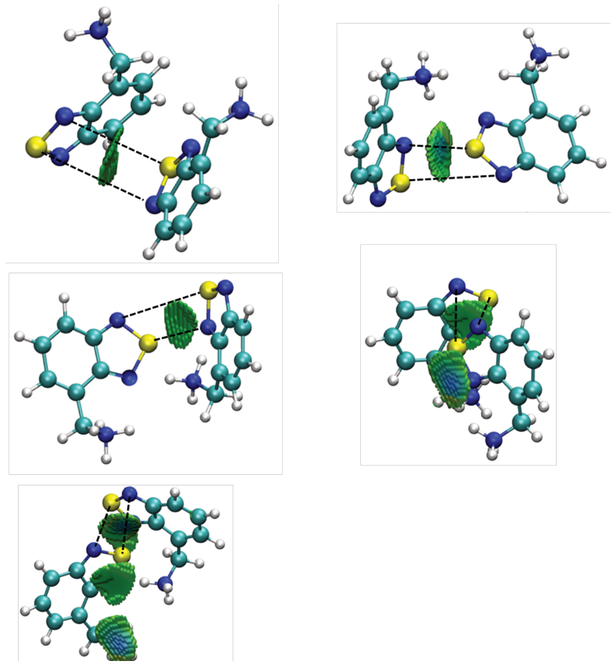

Fig. S4. Examples of intralayer CB formation within the same organic layer from an equilibrated AIMD trajectory of the *rotated interlayer* model. In all cases, CBs are denoted with black broken lines and visualized through NCIPLOT with green isosurfaces indicating weak noncovalent interactions.

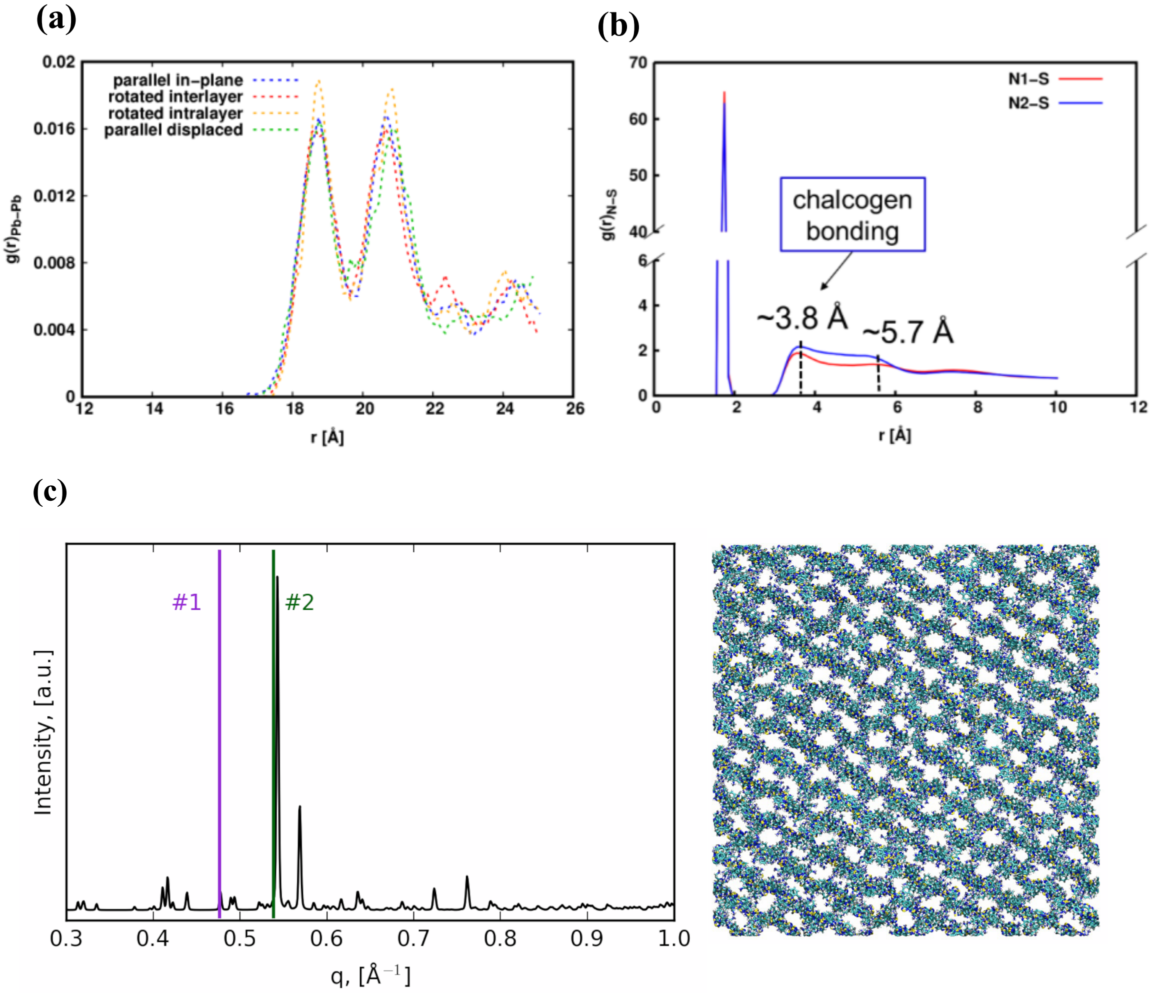

Fig. S5. Radial distribution function g(r) for an n = 1 RP model system between a) Pb atoms belonging to different inorganic layers, b) nitrogen (N_1 and N_2, see Fig. S1) and sulfur atoms. The first peak in part (b) corresponds to the strong covalent bond formed between N and S atoms in a single BTDZ molecule. The second peak, at r = 3.8 Å, is indicative of the formation of CBs between adjacent interacting BTDZ molecules, whereas the third peak, at r = 5.7 Å, describes weaker interactions between the S atom of one molecule and the more distant N atom of the thiadiazole ring of the second molecule. (c) Left: Simulated XRD diffraction pattern of the (BTDZ)PbI_3-based 1D δ-phase after replacing Cs with BTDZ. The three lines indicate the position of the experimental peaks (Fig. S11 and Table S2). Right: Molecular representation of the equilibrated 1D (BTDZ)PbI_3 δ-phase showing the continuous cross-linked network of the organic phase. The face-sharing PbI_6 octahedra are normal to the shown plane and are hidden for clarity.

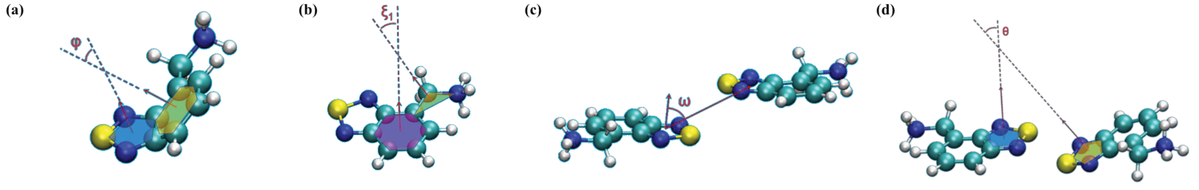

Fig. S6. Schematic for the definition of the four conformational angles: a) the molecule planarity angle φ, b) the substituent rotation angle ξ_1, c) the off-plane angle ω and d) the ring rotation angle θ. In all parts, red arrows denote normal vectors to corresponding planes. In part (c), one red vector connects the two centers of mass of the thiadiazole rings, and the remaining vector indicates the normal to one thiadiazole ring.

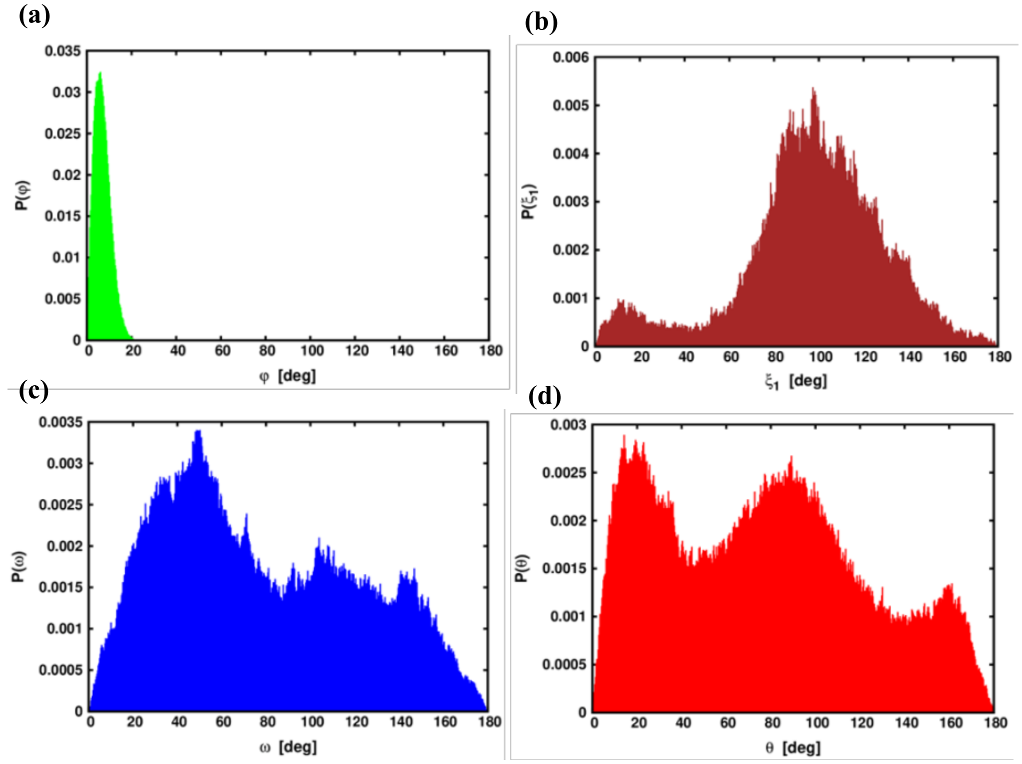

Fig. S7. Histograms of the four conformational angles considered here averaged over the AIMD trajectory for the most stable, *rotated interlayer*, starting configuration. Part (a) shows that the ring system of the BTDZ molecule exhibits increased stiffness with a small bending angle of only 6°, whereas (b) shows that the methylammonium substituent exhibits a dominant rotation angle of about 90° off the ring plane. Parts (c) and (d) summarize the prevalent molecular orientation during CB formation, dictating that the two BTDZ molecules are predominantly out of plane by about 45° and the ring planes are rotated by about 20° with respect to each other. Interestingly, a secondary smaller peak at 90° is observed in part (d) which corresponds to the perfect T-shaped π-stacking of the two BTDZ molecules. The third peak appearing at 160° is the supplementary angle of the first peak at 20°.

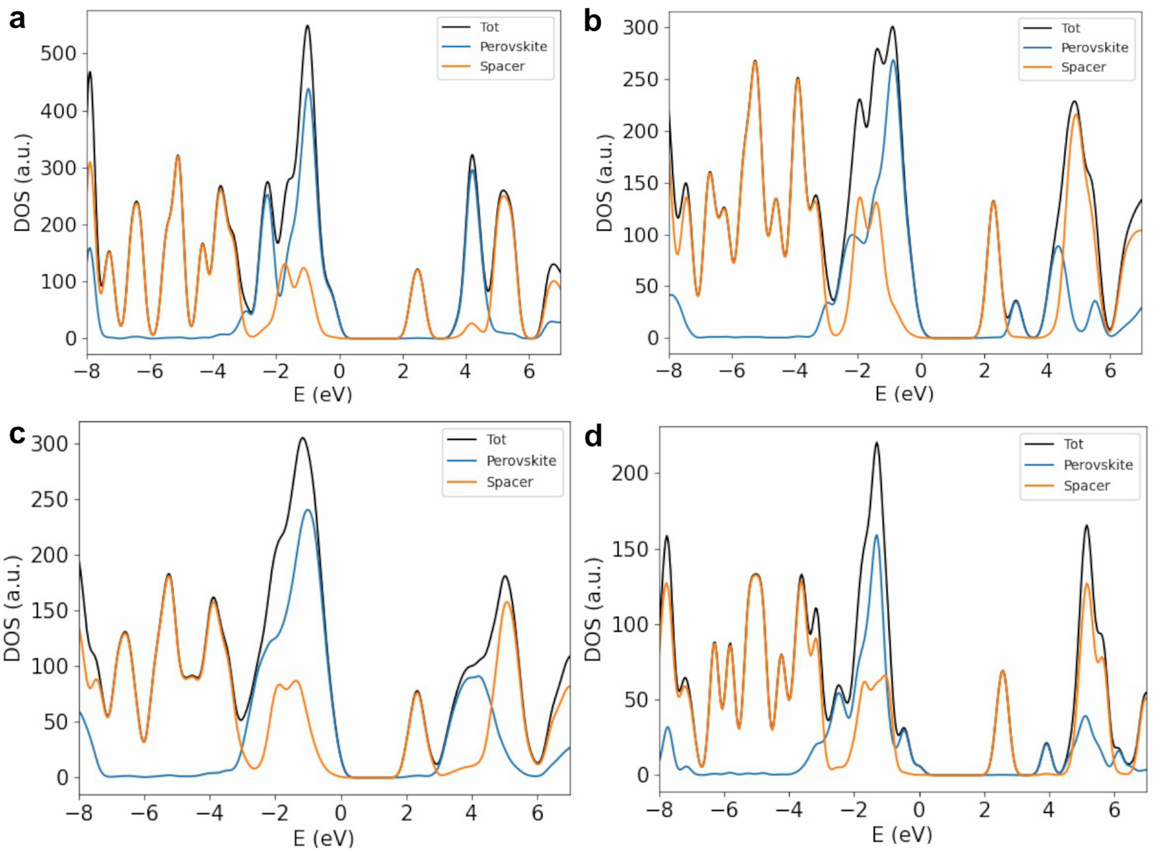

Fig. S8. Calculated PDOS for a) 1D $BTDZPbI_3$, b) 2D $(BTDZ)_2PbI_4$, c) δ-$BTDZPbI_3$, d) 2D $(BTDZ)_2PbBr_4$. Based on the PDOS, 2D $(BTDZ)_2PbI_4$ and the corresponding 1D δ-phase systems could contribute favourably to supporting charge transfer as the molecular orbitals of BTDZ are overlapping with the bottom of the perovskite conduction band, reducing the band gap. The remaining systems, 1D $(BTDZ)PbI_3$ and 2D $(BTDZ)_2PbBr_4$, exhibit trapped states within the band gap from the BTDZ spacer, inhibiting charge transfer.

Table S1. Relative formation energies per stoichiometric unit of all LD systems considered in this study. Energies are given at 0 K in reference to the most stable, i.e., $(BTDZ)_2PbBr_4$, LD system.

**LD System**	E_f **[eV]**
1D δ-$(BTDZ)PbI_3$	6.47
1D $(BTDZ)PbI_3$	5.90
2D $(BTDZ)_2PbI_4$ parallel in-plane	1.87
rotated interlayer	1.82
rotated intralayer	1.81
parallel displaced	1.91
2D $(BTDZ)_2PbBr_4$ S–π stabilized	0.00
CB-stabilized parallel in-plane	0.31

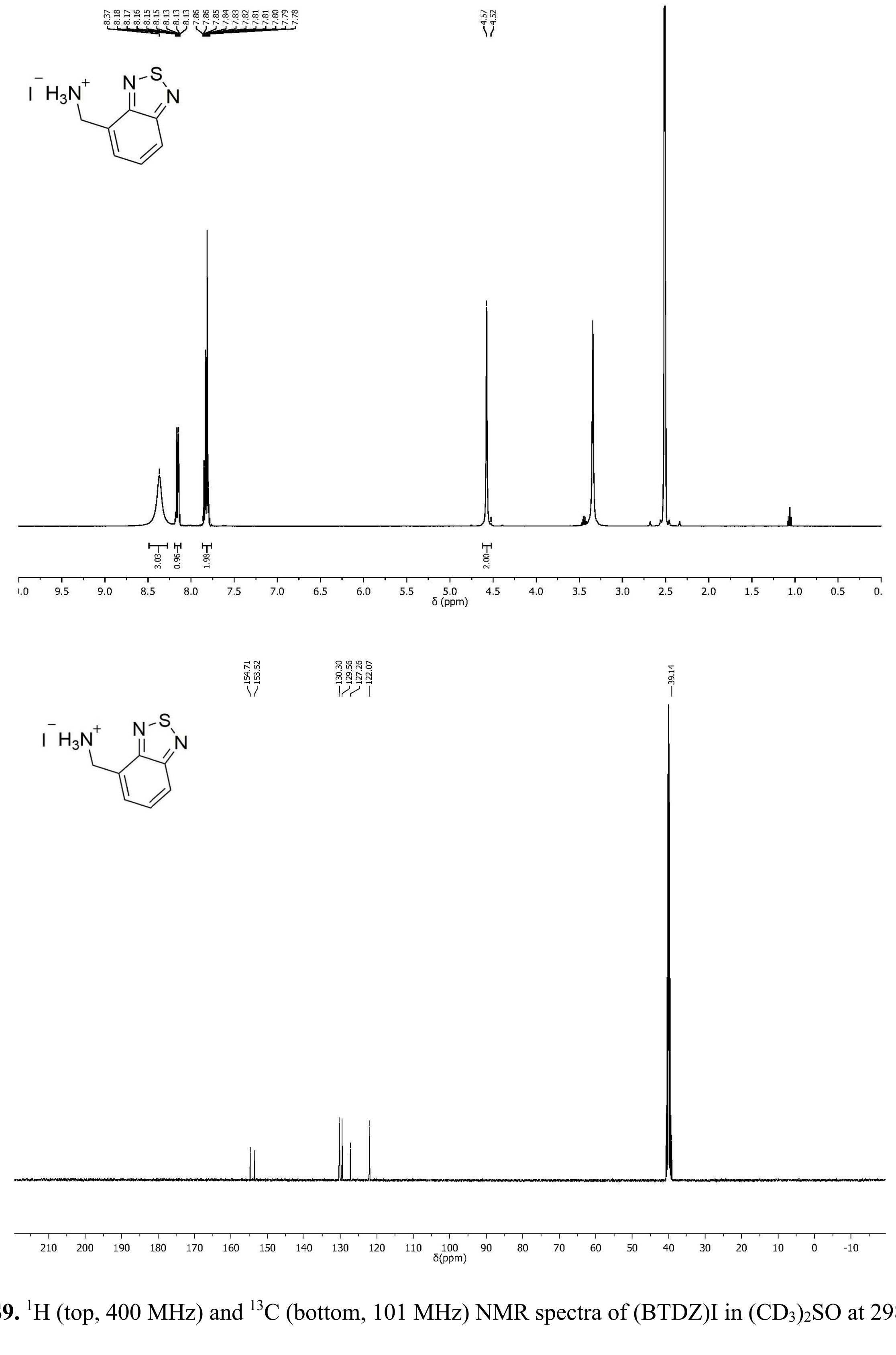

Fig. S9. ^{1}H (top, 400 MHz) and ^{13}C (bottom, 101 MHz) NMR spectra of (BTDZ)I in $(CD_3)_2SO$ at 298 K.

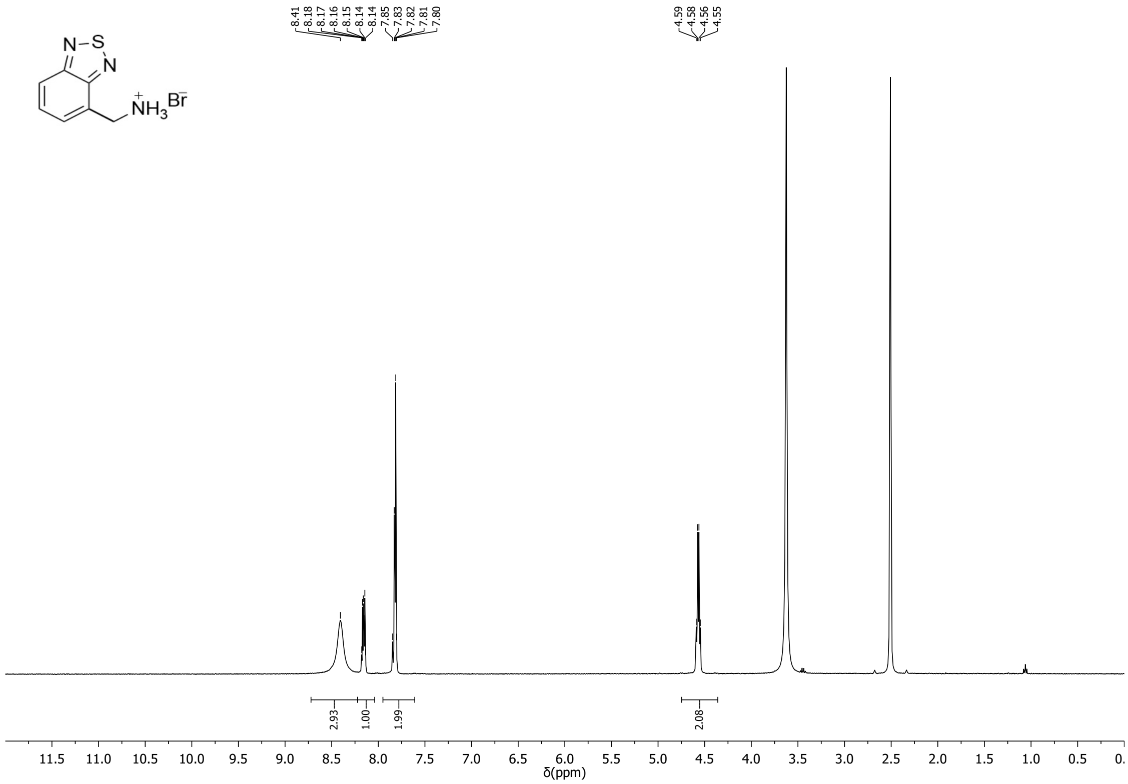

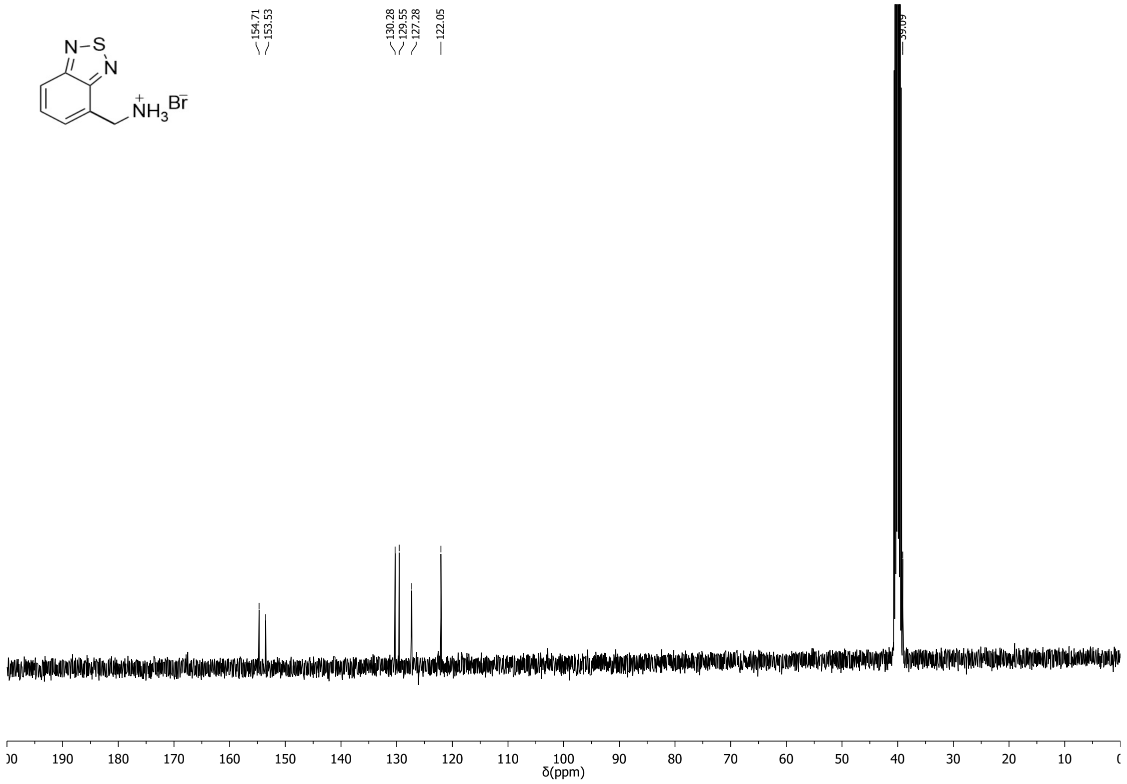

Fig. S10. ^{1}H (top, 400 MHz) and ^{13}C (bottom, 101 MHz) NMR spectra of (BTDZ)Br in $(CD_3)_2SO$ at 298 K.

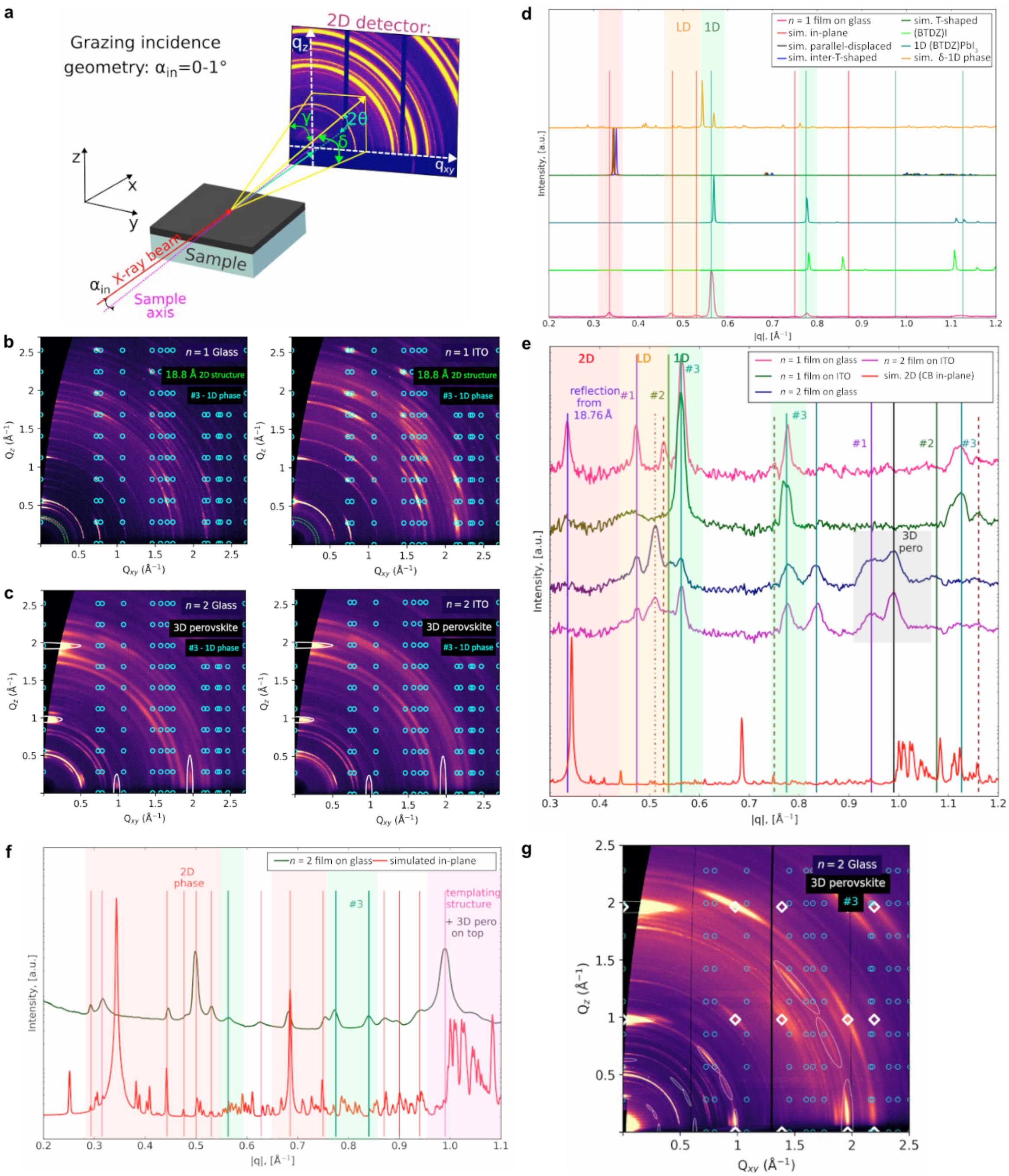

Fig. S11. (a) GIWAXS measurements setup and (b–c) reciprocal space maps measured at 0.3° incidence angle, probing the whole thickness of the (BTZD)I-based films with marked structures, namely 2D phase with CB (green circles), 1D (BTDZ)PbI_3 (cyan circles), and 3D perovskite (α-$FAPbI_3$ phase, white ovals) in (b) $n = 1$ and (c) $n = 2$ $(BTDZ)_2FA_{n-1}Pb_nI_{3n+1}$ nominal compositions on glass (left) and ITO (right). The out-of-plane orientation of the 3D perovskite phase (white ovals) appears independently of the substrate. (d–f) A comparison of angular profiles extracted from GIWAXS reciprocal space maps (in b, left and g) and powder diffraction from the simulated structures. (g) GIWAXS reciprocal space map measured at 0.3° incidence angle, probing the whole thickness of the films with marked structures, namely $n = 2$ 2D structure (20 Å, white ovals), (BTDZ)PbI_3 (cyan circles), and 3D perovskite (α-$FAPbI_3$ phase, white diamonds and ovals > 1 $Å^{-1}$) in $n = 2$ $(BTDZ)_2FAPb_2I_7$ nominal composition on glass. Bragg peak positions and corresponding structures are listed in Table S2–S3 and the details are included in the Supplementary Discussion section.

Table S2. List of Bragg peaks from LD perovskite structures based on GIWAXS profiles of n = 1 nominal composition on glass (Fig. S11d). #1–2 indicate other LD phases identified in GIWAXS profiles.

Bragg Peak Position	Structure
0.334 $Å^{-1}$	2D structure (corresponding to CB 2D in-plane)
0.476 $Å^{-1}$	LD structure #1
0.53 $Å^{-1}$	LD structure #2
0.563 $Å^{-1}$	(BTDZ)PbI_3 1D phase
0.75 $Å^{-1}$	LD structure
0.775 $Å^{-1}$	(BTDZ)PbI_3 1D phase
0.84 $Å^{-1}$	(BTDZ)PbI_3 1D phase
0.94 $Å^{-1}$	LD structure #1

Table S3. List of Bragg peaks from LD and 3D perovskite structures based on GIWAXS profiles of n = 2 nominal composition on glass (Fig. S11e–f). #1–2 indicate other LD phases identified in GIWAXS profiles.

Bragg Peak Position	Structure
0.293 $Å^{-1}$	LD structure
0.315 $Å^{-1}$	2D n = 2 phase (oriented)
0.443 $Å^{-1}$	LD structure
0.5 $Å^{-1}$	LD structure #1
0.53 $Å^{-1}$	LD structure #2
0.563 $Å^{-1}$	(BTDZ)PbI_3 1D phase
0.63 $Å^{-1}$	2D n = 2 phase (oriented)
0.685 $Å^{-1}$	LD structure
0.75 $Å^{-1}$	LD structure #2
0.775 $Å^{-1}$	(BTDZ)PbI_3 1D phase
0.835 $Å^{-1}$	2H hexagonal δ-$FAPbI_3$ and (BTDZ)PbI_3 1D phase
0.86 $Å^{-1}$	6H hexagonal $FAPbI_3$
0.89 $Å^{-1}$	LD structure and PbI_2
0.94 $Å^{-1}$	LD structure #1
0.99 $Å^{-1}$	Templated 3D perovskite (the same orientation as 2D n = 2 phase)
1.04 $Å^{-1}$	2D n = 2 phase (oriented)

Table S4. Selected crystal data and refinement parameters for (BTDZ)I, (BTDZ)PbI_3 and $(BTDZ)_2PbBr_4$.

Chemical formula	$[C_7H_8N_3S]I$	$[C_7H_8N_3S]PbI_3$	$[C_7H_8N_3S]_2PbBr_4$
Formula Mass	293.12	1508.23	859.28
Crystal system	monoclinic	orthorhombic	orthorhombic
a (Å)	8.102(3)	7.850(3)	7.918(3)
b (Å)	8.591(3)	8.648(3)	34.823(6)
c (Å)	13.931(4)	22.049(6)	8.405(3)
β (°)	98.77(3)		
Unit cell volume (Å^3)	958.3(6)	1496.8(9)	2317.5(13)
Temperature (K)	100(2)	100(2)	100(2)
Space group	$P2_1/c$	$P2_12_12_1$	*Pnma*
No. of formula units per unit cell, *Z*	4	2	4
No. of reflections measured	10270	11223	16375
No. of independent reflections	1949	2921	2413
R_{int}	0.0266	0.0353	0.0567
Final R_1 values ($I > 2\sigma(I)$)	0.0200	0.0229	0.0325
Final $wR(F^2)$ values ($I > 2\sigma(I)$)	0.0501	0.0571	0.0828
Final R_1 values (all data)	0.0218	0.0235	0.0328
Final $wR(F^2)$ values (all data)	0.0508	0.0574	0.0831

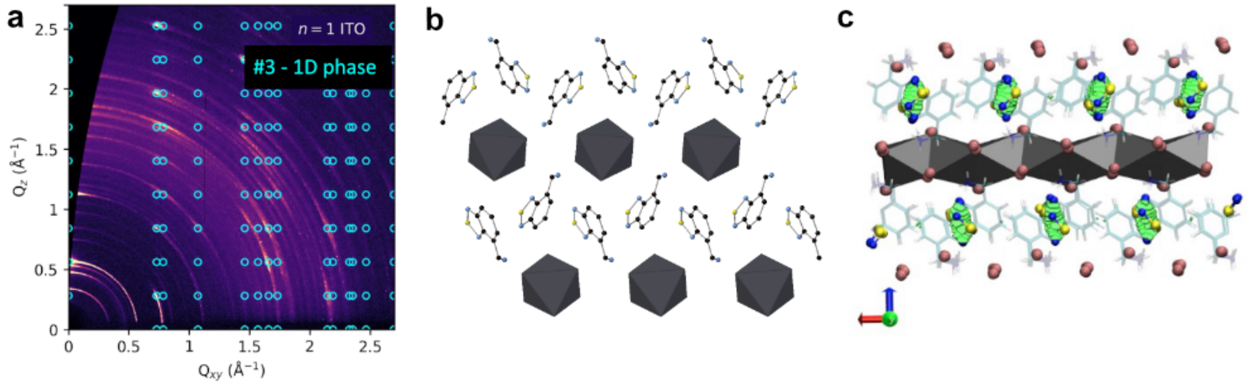

Fig. S12. (a) GIWAXS pattern of (BTDZ)PbI_4 $n = 1$ films on ITO with a fitted 1D structure #3 (a = 8.6 Å, b = 8 Å, c = 22.4 Å, $\alpha = \beta = \gamma = 90°$). Light blue circles mark the expected peak positions for the fitted structure. (b) Crystal structures of 1D (BTDZ)PbI_3 phase, (c) with interactions visualized in green with NCIPLOT.

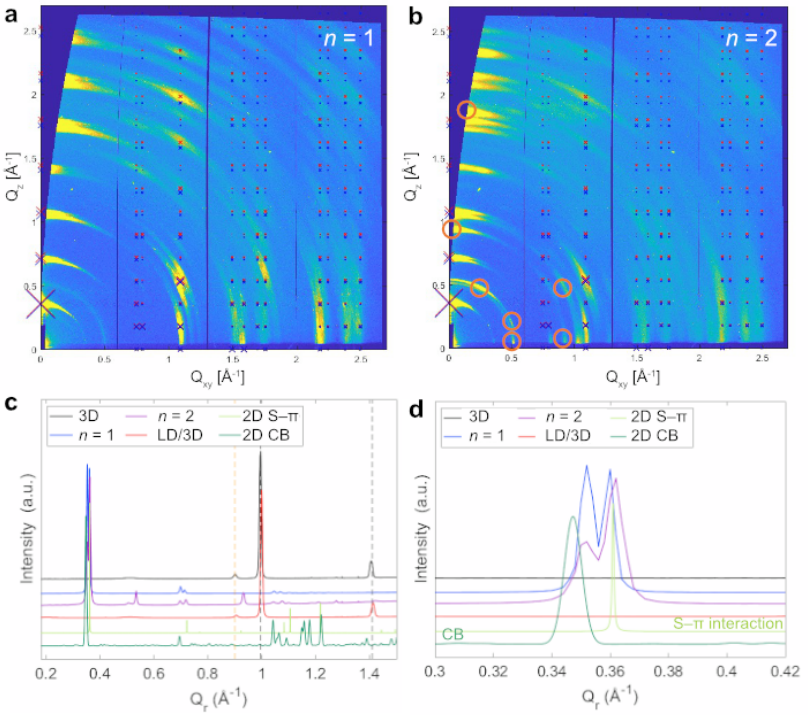

Fig. S13. (a–b) GIWAXS reciprocal space maps measured at 0.3° incidence angle for $(BTDZ)_2PbBr_4$ (n = 1) and $(BTDZ)_2FAPb_2Br_7$ (n = 2) nominal compositions. Red markers: $(BTDZ)_2PbBr_4$ layered structure (based on the crystal structure stabilizsd by S-π interactions) in an orientation with the layers parallel to the substrate. Blue markers: a similar structure with an extended c-axis (i.e., stacking distance), corresponding to the layered structure stabilised by CB. Orange circles: unidentified peaks, likely corresponding to 1D or 2D n = 2 phases. (c–d) Comparison of radial profiles extracted from GIWAXS reciprocal space maps (IN black, blue, purple, and red) and simulated diffraction patterns (in green) of layered structures stabilised by S-π and CB interactions. A close-up of the 2D phase signal is shown in d), showing a double peak, indicating two different structures corresponding to CB and S–π interactions. Control is a reference 3D perovskite thin film, while Br-LD/3D indicates perovskite thin films treated with (BDTZ)Br.

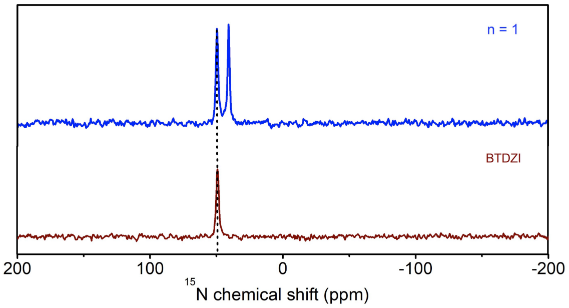

Fig. S14. ^{15}N CP NMR spectra of the (BTDZ)I and corresponding mechanosynthesised n = 1 2D perovskite recorded at 20 T (12 kHz MAS) at room temperature. Note that the material after mechanosynthesis contains a mixture of n = 1 and unreacted (BTDZ)I. Acquisition parameters were as follows. (BTDZ)I: recycle delay 1 s, number of scans 10000; n = 1: recycle delay 1 s, number of scans 57548. Lorentzian broadening of 50 Hz was applied to both spectra. A contact time of 1 ms was used for the CP.

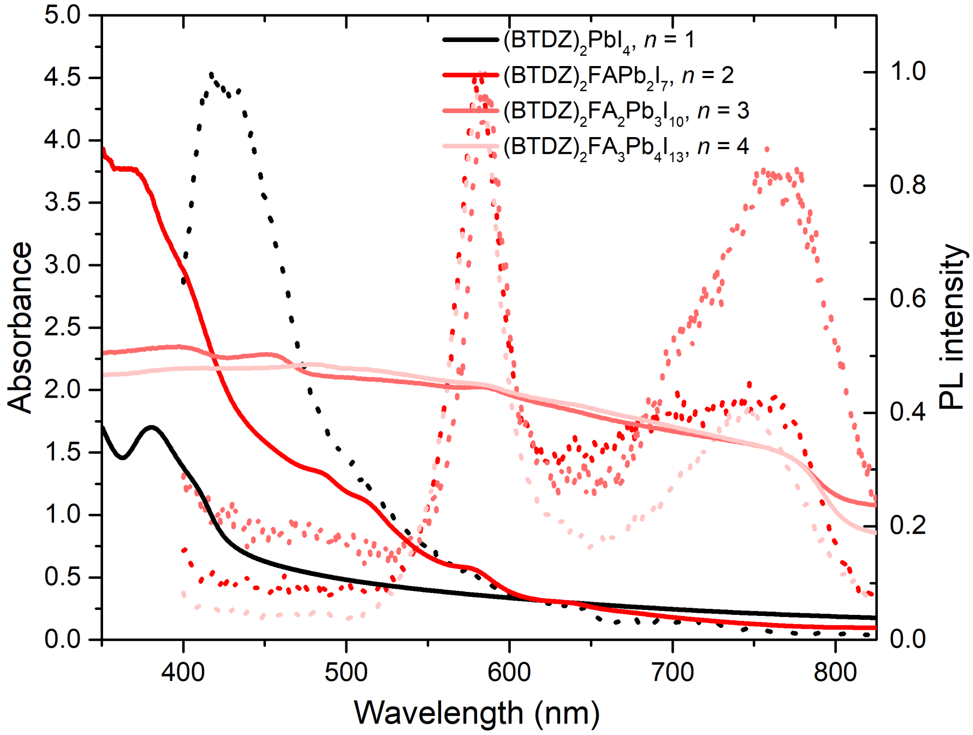

Fig. S15. UV-vis absorption (full lines, left) and PL (dashed lines, right) spectra of BTDZI-based perovskite films of nominal n = 1–4 composition.

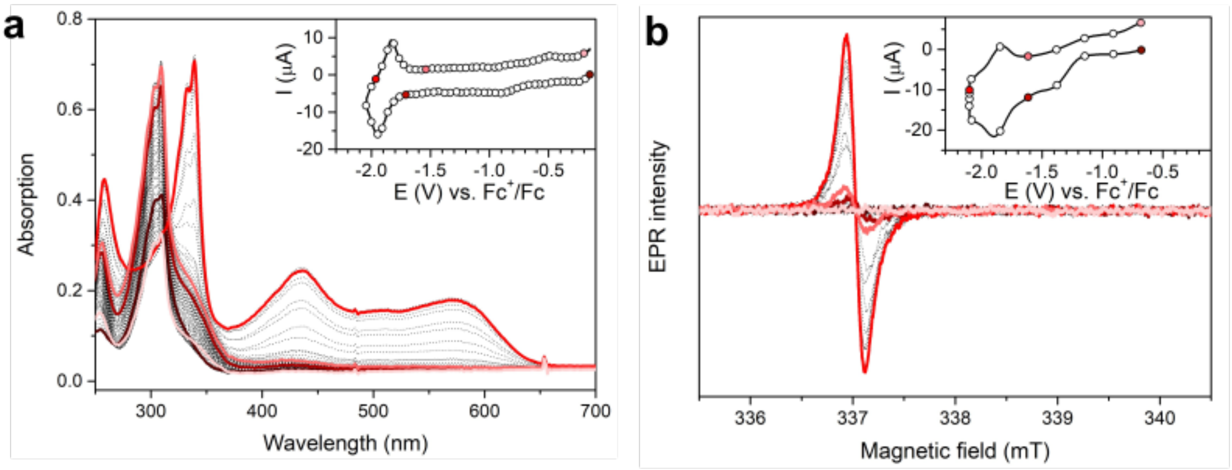

Fig. S16. (a) UV-vis SEC record of the (BTDZ)I spacer precursor in solution during the cathodic CV scan shown in the inset; coloured circles signify potentials at which identically coloured UV-vis spectra were recorded. (b) EPR/vis-SEC record of the organic spacer precursor solution. Spectra were recorded during the cathodic CV scan shown in the inset, with the coloured circles corresponding to the potentials identically coloured in UV-vis absorption spectra (with Pt mesh working electrode 3 mVs^{-1} scan rate). After the forward cathodic scan, the potential was maintained at -2.1 V for 5 min to accumulate the generated paramagnetic species before re-oxidation anodic sweep. Single line with g_{iso} of 2.0048 ± 0.0002 and ΔB_{pp} of 0.18 mT.[]

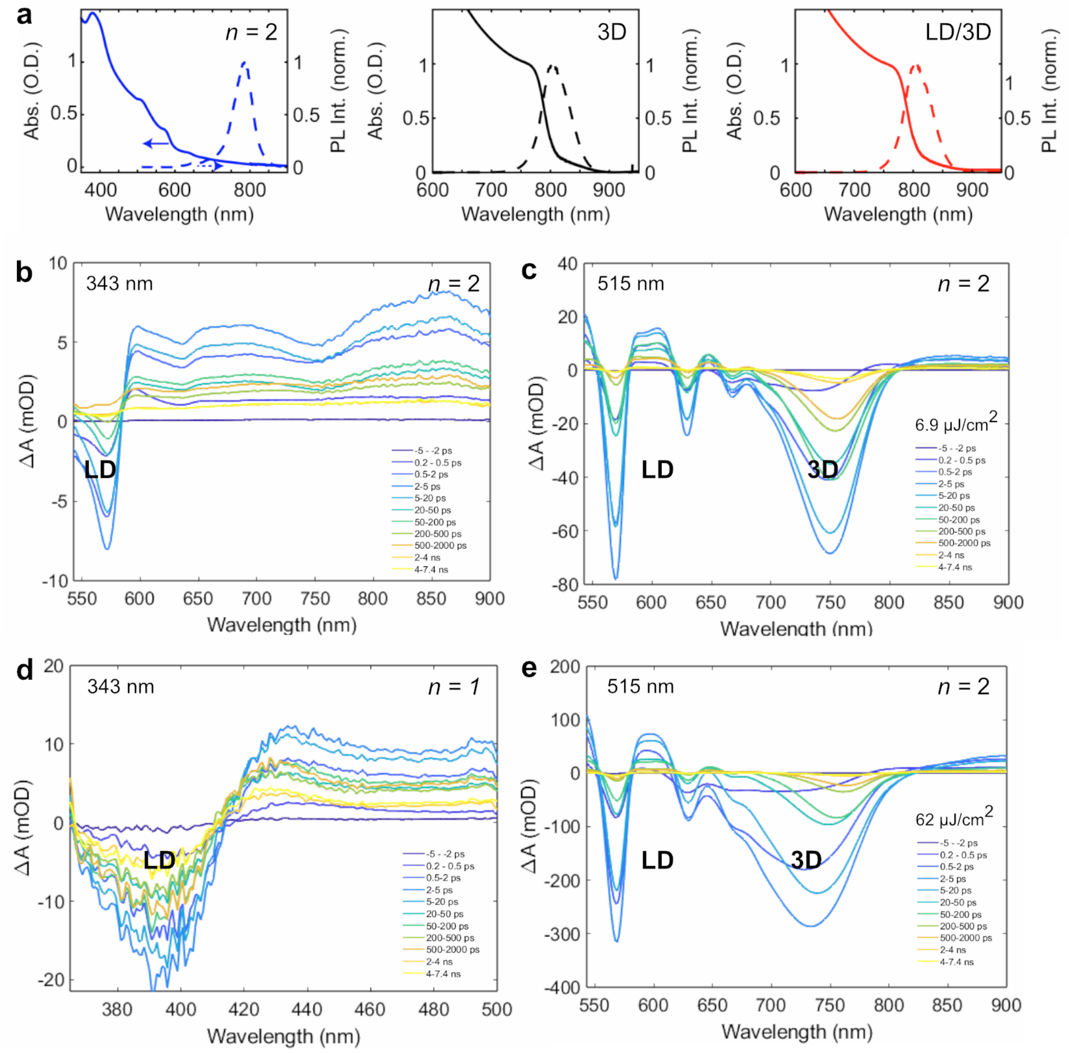

Fig. S17. (a) UV-vis absorption and photoluminescence (PL, dashed lines) spectra of thin films of $(BTDZ)_2FAPb_2I_7$ (n = 2, blue) nominal composition, 3D (black), and mixed LD/3D perovskites (red) on microscope glass. (b–e) Transient absorption spectra of films of (b–c,e) $(BTDZ)_2FAPb_2I_7$ (n = 2) nominal composition at varying time delays and fluence (6.9 μJ/cm^2 for b,c and 63 μJ/cm^2 for e) upon excitation at 343 nm (b) and 515 nm (c,e), indicating characteristic (bleach) signals corresponding to LD and 3D phases.

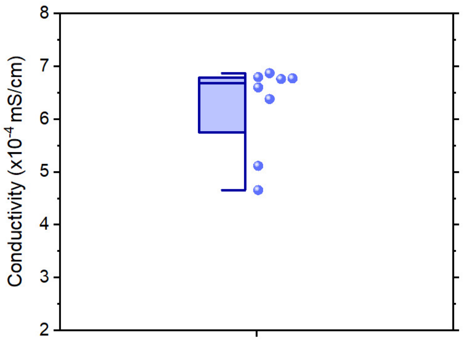

Fig. S18. Lateral conductivity measurements performed on 8 devices of $(BTDZ)_2PbI_4$ nominal composition.

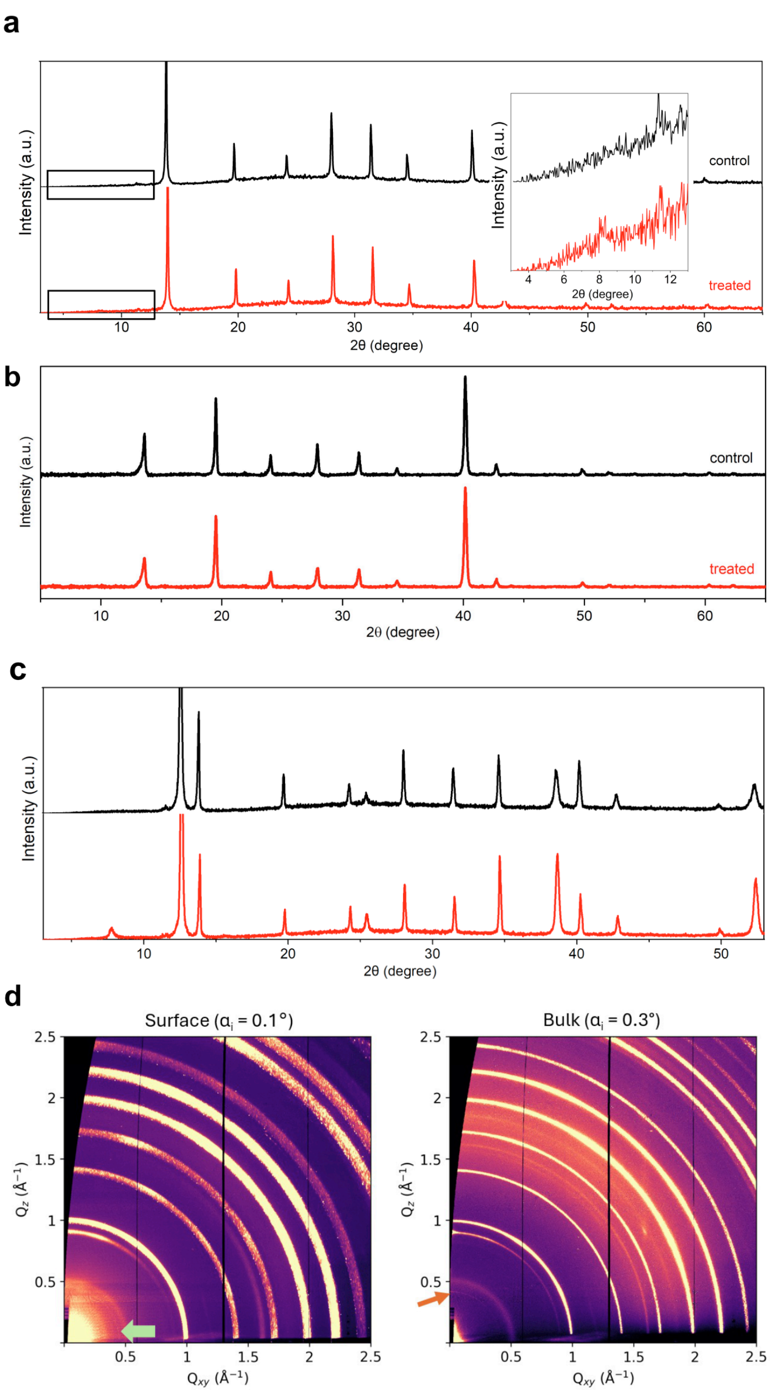

Fig. S19. XRD pattern of (a) 3D perovskite films without (control) and with (treated) BTDZ overlayer and (b) 3D perovskite films without (control) and with (treated) (BTDZ)I, highlighting the low-angle reflection peak at 8 degrees (a, inset) that is more apparent in higher concentrations of (BTDZ)I (5 mg/mL in c). (d) GIWAXS reciprocal space map of 3D perovskite films with (BTDZ)I overlayer measured at (left) 0.1° and (right) 0.3° incidence angle, probing the surface and the whole thickness of the film, respectively. Bragg peaks at 0.45 $Å^{-1}$ (surface) and 0.42 $Å^{-1}$ (whole sample) correspond to LD phases on top of 3D perovskite.

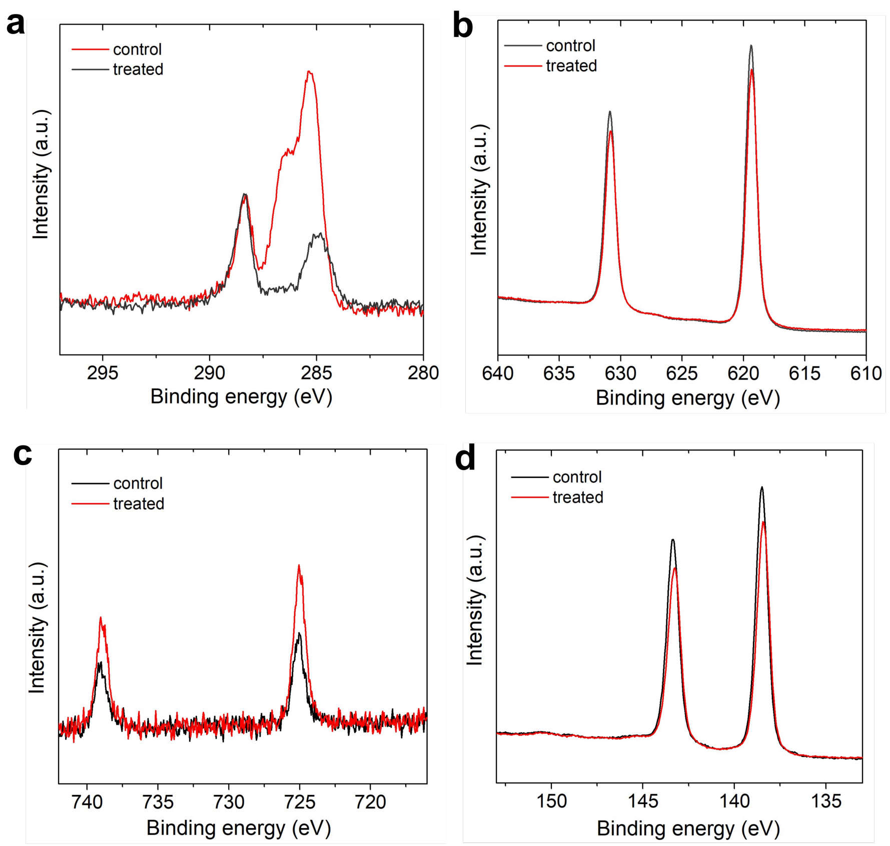

Fig. S20. (a) C 1*s*, (b) I 3*d*, (c) Cs 3*d*, (d) Pb 4*f* XPS spectra measured on reference and BTDZ-treated films.

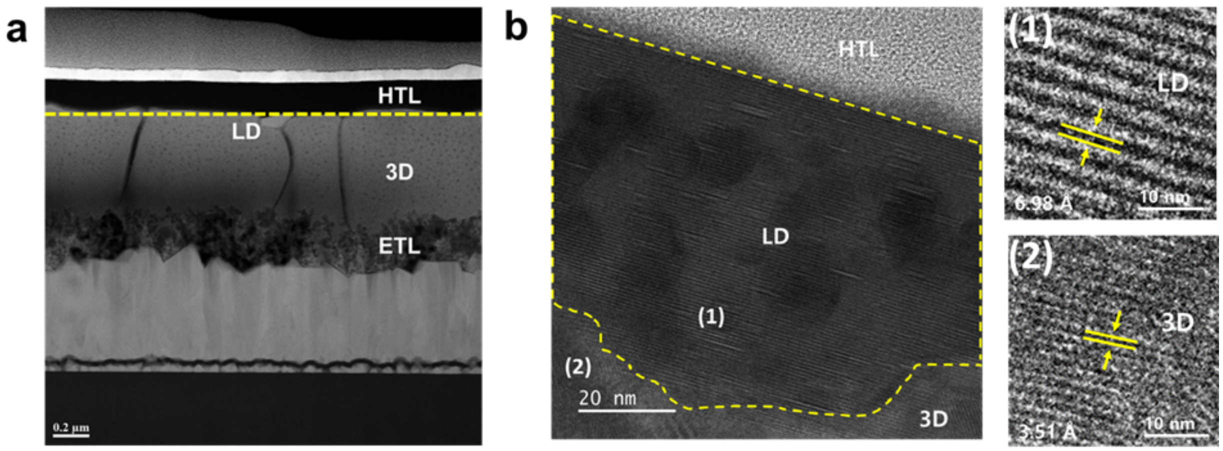

Fig. S21. Cross-sectional STEM image of perovskite solar cell with BTDZ and (b) TEM images of the perovskite film with BTDZ, highlighting the co-existence of 3D and low-dimensional (LD) perovskite phases at the interface with the hole-transport material (HTM).

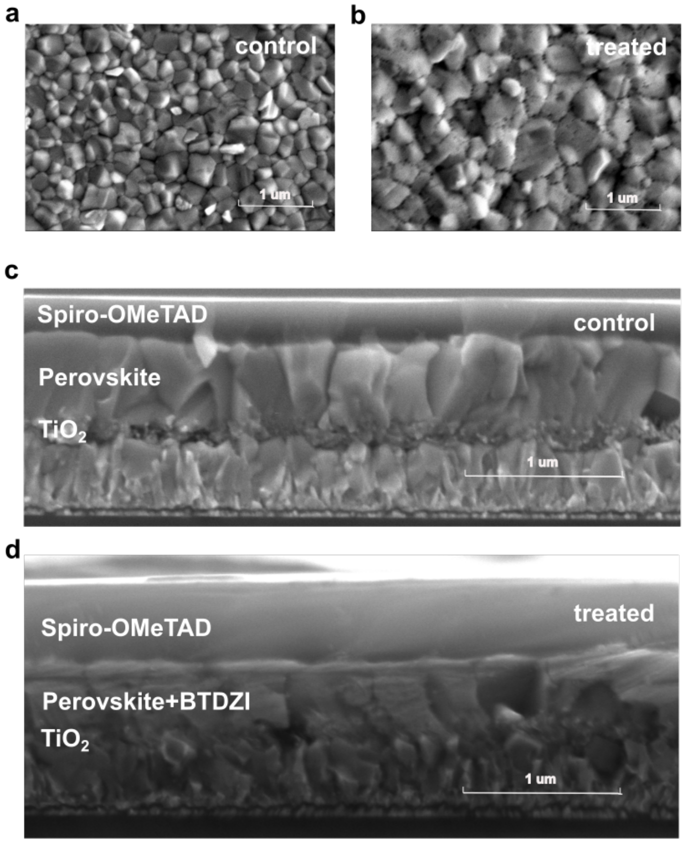

Fig. S22. (a) SEM images of 3D perovskite films surface (a) without (control) and (b) with (treated) (BTDZ)I overlayer, forming LD/3D perovskite films. (c–d) SEM cross-section image of the n-i-p perovskite solar cell (c) without (control) and (d) with (treated) (BTDZ)I overlayer forming LD/3D perovskite films.

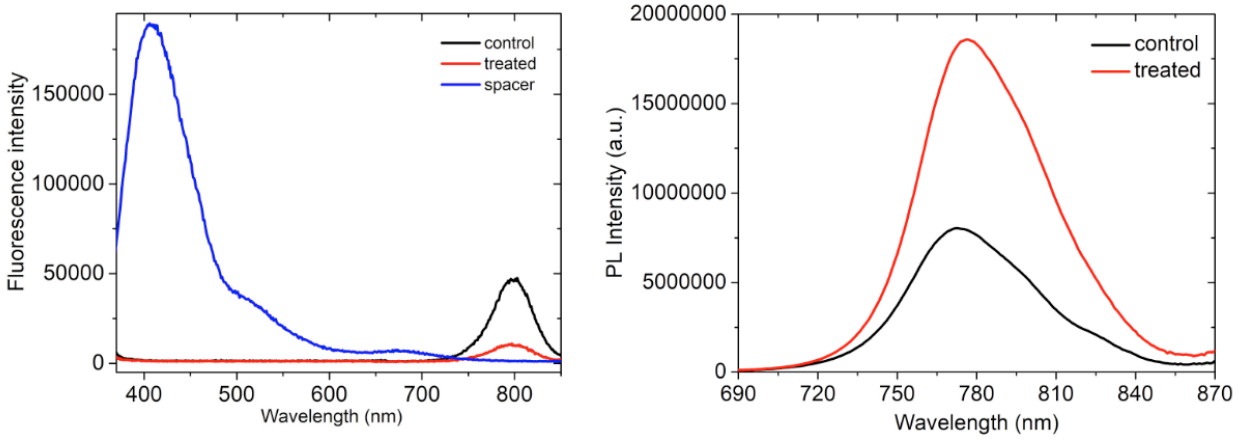

Fig. S23. Left: PL spectra of 3D perovskite films without (control, black) and with (treated, red) (BTDZ)I overlayer, forming LD/3D perovskite films, compared to neat (BTDZ)I films (blue). Right: PL spectra of 3D perovskite thin films without (control) and with (treated) (BTDZ)I additive to 3D perovskite composition.

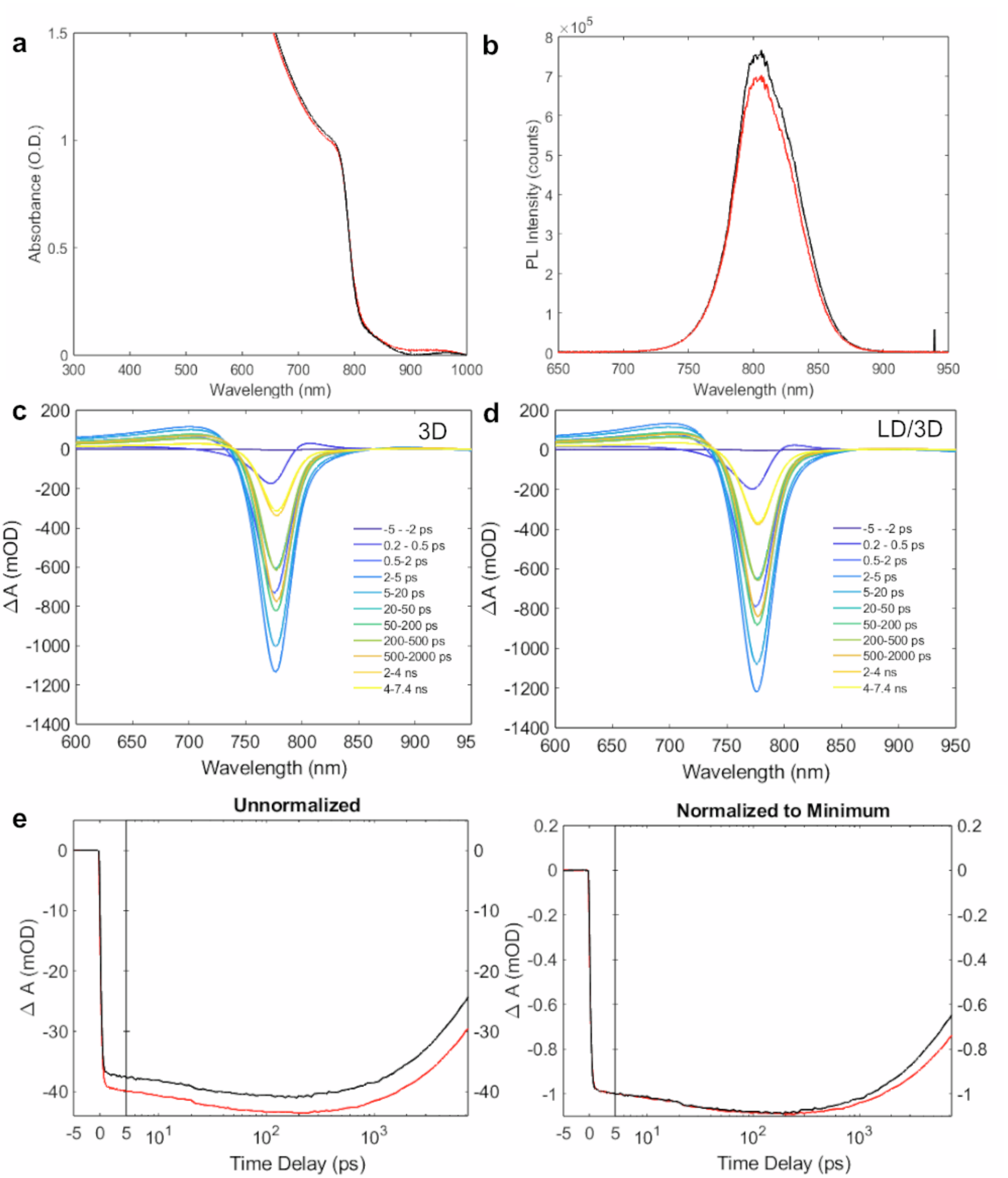

Fig. S24. (a) UV-vis absorption and (b) photoluminescence (PL, dashed lines) emission spectra of 3D (black), and mixed LD/3D (red) perovskite thin films on microscope glass. (c–d) Transient absorption spectra of 3D (f) and (g) mixed LD/3D perovskite thin films on microscope glass at varying time delays upon excitation at 515 nm (6.9 mJ/cm^2 fluence). (e) Kinetics from the ground state bleach at 777 nm, highlighting faster recovery of the 3D perovskite (black) as compared to the mixed LD/3D system (red) that can be associated with a passivation effect of the LD overlayer on the 3D perovskite thin films.

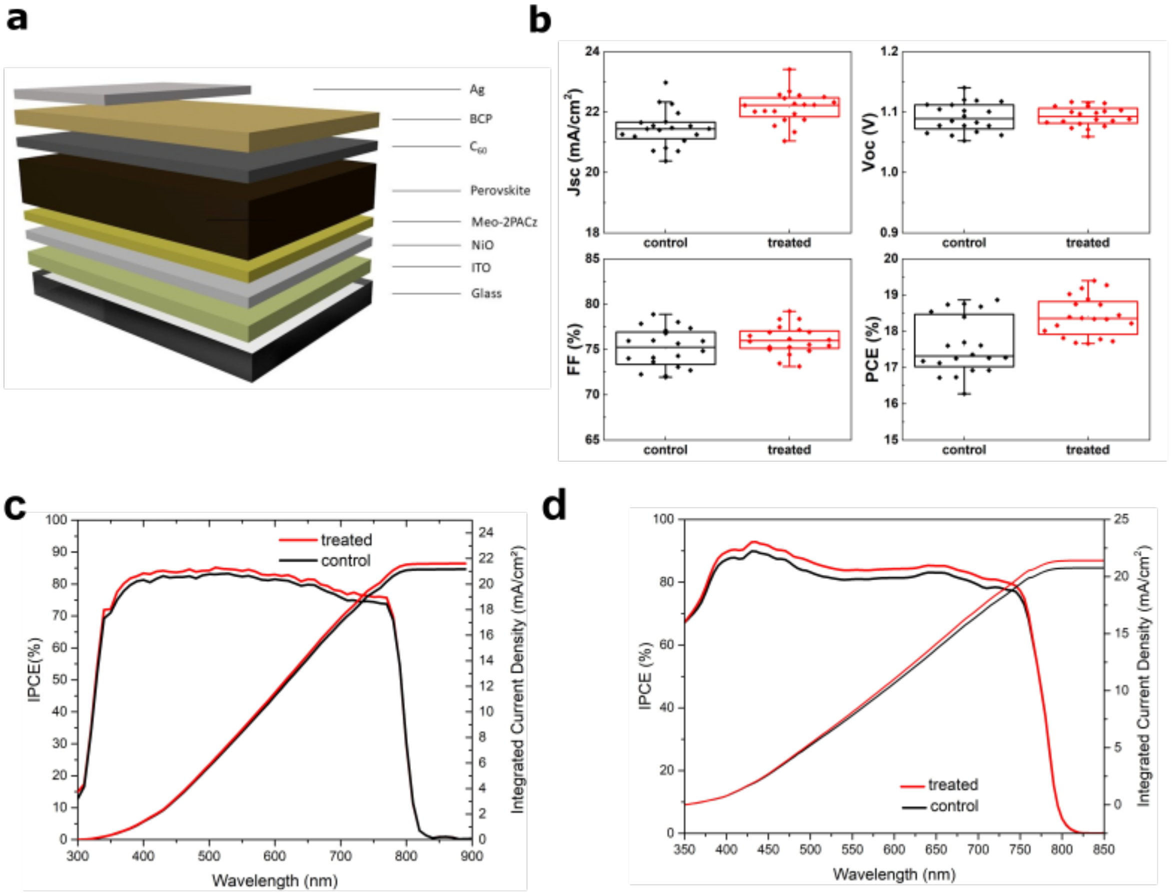

Fig. S25. (a) Schematic of p-i-n perovskite solar cell architecture and the corresponding (b) box charts illustrating the statistical distribution of photovoltaic parameters: open-circuit voltage (V_{OC}), short-circuit current density (J_{SC}), fill factor (FF), and power conversion efficiency (PCE). (c–d) IPCE spectra of the (c) n-i-p and (d) p-i-n perovskite solar cells without (control, black) and with (treated, red) (BTDZ)I.

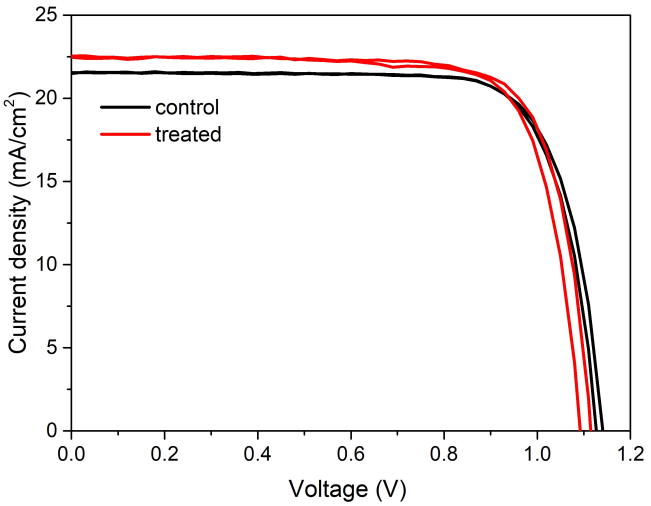

Fig S26. Current-voltage characteristic of perovskite solar cells treated with and without (BTDZ)I.

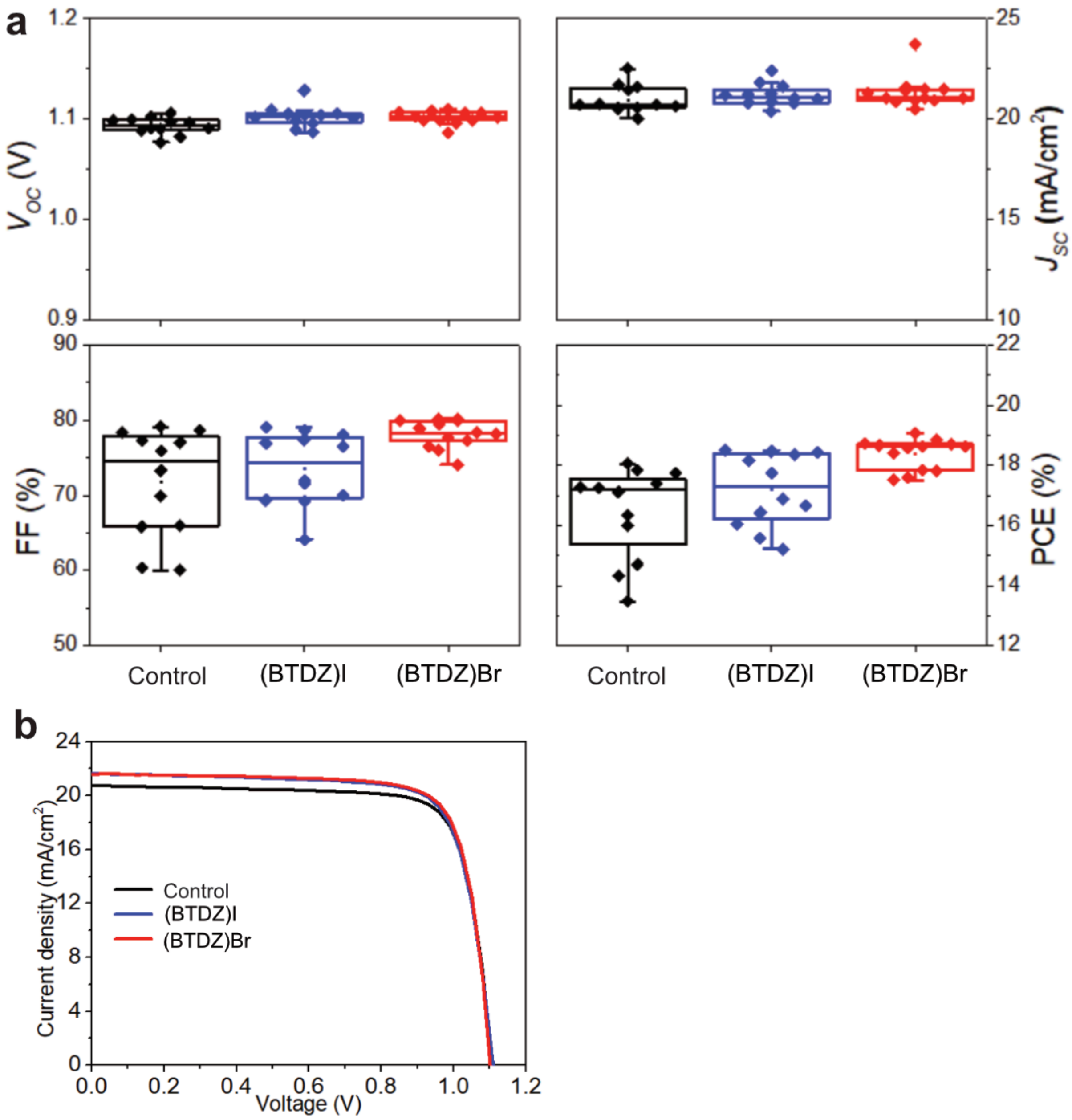

Fig. S27. (a) Box charts illustrating the statistical distribution of photovoltaic parameters upon (BTDZ)Br (red) and (BTDZ)I (blue) treatment as compared to the control (black) in p-i-n device architectures. (b) Current-voltage characteristics of the champion devices for upon (BTDZ)Br (red) and (BTDZ)I (blue) treatment as compared to the control (black) in p-i-n device architectures. The results suggest improvements in short-circuit currents and fill factor for (BTDZ)Br systems, which could be associated with the higher contribution of the 2D perovskite phase for bromide-based perovskite as compared to iodide compositions.

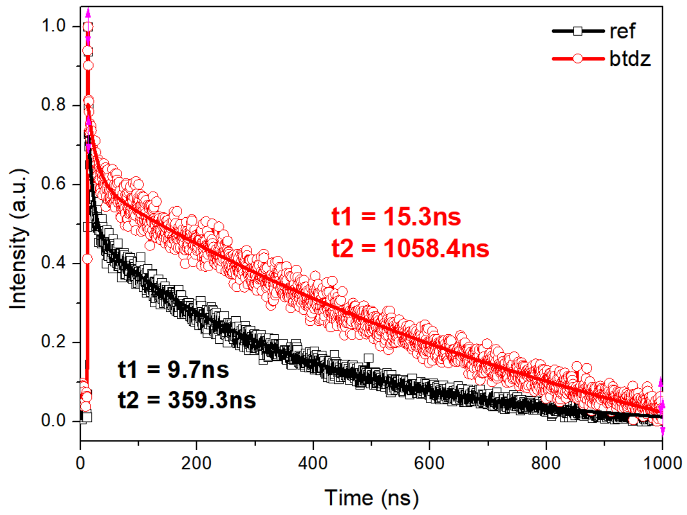

Fig. S28. Time-resolved PL of the 3D perovskite films (ref) with and without (BTDZ)I overlayer.

Table S5. Summary of the calculated lifetime for the perovskite films with and without (BTDZ)I treatment.

	Equation	t_1 /ns	t_2 / ns
Control	$y = y_0 + A_1 e^{-\frac{(x-x_0)}{t_1}} + A_2 e^{-\frac{(x-x_0)}{t_2}}$	9.7	359.3
Treated		15.3	1058.4

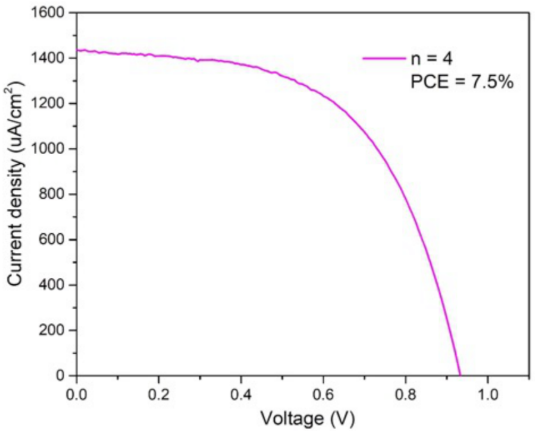

Fig. S29. J–V curve of BTDZ-based perovskites of n = 4 nominal compositions in n-i-p devices under AM 1.5G illumination. While further optimisation of the film fabrication, phase purity, and device architecture could contribute to enhancing the device performances, the photovoltaic performances of n = 4 nominal compositions compare to some of the best-performing MA-free FA-based quasi-2D perovskite solar cells using mp-TiO_2 to date.[22–23]

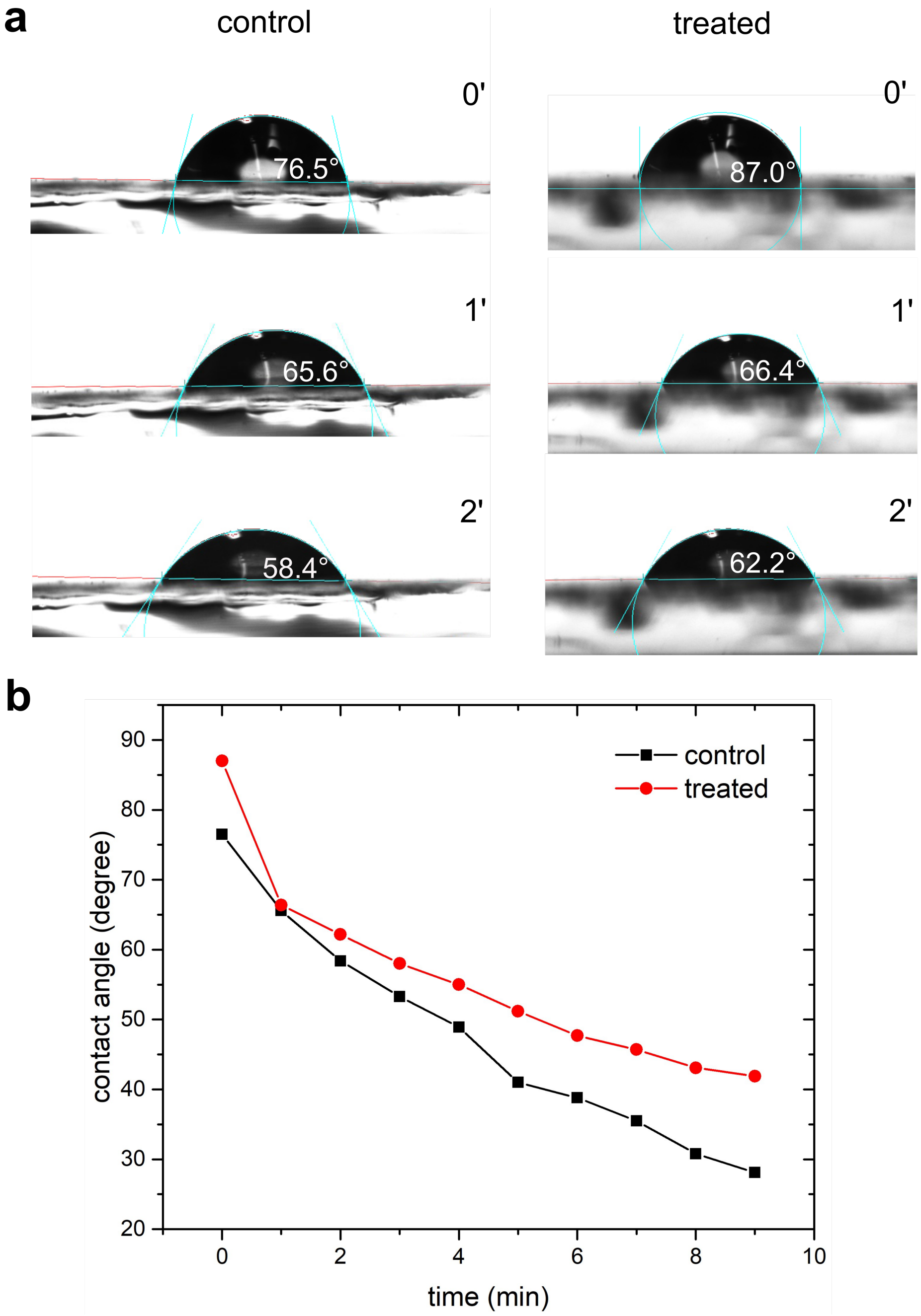

Fig. S30. (a) Contact angle of the water droplet on top of the 3D perovskite film without (control, left) and with (treated, right) (BTDZ)I overlayer. (b) Scatter plot of the corresponding contact angles (in a) as a function of the loading time of the water droplet on top of the 3D perovskite film without (control, black) and with (treated, red) (BTDZ)I overlayer in LD/3D perovskite films.

Supplementary Discussion

GIWAXS measurements (Fig. S11a) were performed to identify low-dimensional (LD) structures that co-exist in the thin films based on n = 1–2 (X = I, Br) nominal compositions (Fig. S11–S13; Tables S2–S3).

Films of $(BTDZ)_2PbI_4$ nominal n = 1 composition on glass showed LD phases that correspond to the simulated 2D CB structures (Fig. S5), as well as additional signals with first and second-order reflections (labelled as #1 and #2) that feature a similar orientation to the 2D phases. The dominant LD phase was found to be 1D $(BTDZ)PbI_3$ face-sharing phase (Fig. S12 and Table S4). The 2D phase orientations were more random on ITO for n = 1–2 compositions as compared to glass (Fig. S11d–f) and they were more preferred for 3D perovskite phase that co-exists within the n = 2 nominal compositions, which also feature templated 2D/3D phases on glass (Fig. S11f and Fig.S11g). The films of n = 2 nominal compositions exhibit a co-existing 1D phase that corresponds to the face-sharing 1D phase, LD phases that correspond to the simulated 1D δ-phase, and a 2D phase of n = 1 composition, as well as a distinct 2D phase with multiple orientations. The dominant LD polymorph can be observed at 0.51 $Å^{-1}$in samples with n = 2 composition co-existing with LD phases (Fig. S11c), or at 0.5 $Å^{-1}$in the with n = 2 composition and n = 2 2D phase (Fig. S11g). The LD/3D films (i.e., 3D films treated by (BTDZ)I in Fig.S19d) show two distinct Bragg peaks, at 0.42 $Å^{-1}$ (also observed for the 3D films treated by (BTDZ)Br) and at 0.45 $Å^{-1}$, which coincided with an LD phase, observed for the n = 2 composition (Fig. S11g). This indicates the presence of LD phases upon overlayer treatment of the 3D perovskite phase, as further evidenced by TEM (Fig. S21 and Fig. S22).

Films of $(BTDZ)_2PbBr_4$ nominal n = 1 composition on glass showed the presence of a dominant 2D phase that matches the S–π-stabilized 2D perovskite structure identified in the single crystal (Fig. S13, Table S4). The films of n = 2 nominal composition featured additional LD phases that are likely to correspond to the 2D structures, which can also be observed in LD/3D films (i.e., upon treatment of 3D films by (BTDZ)Br). The co-existing 2D phase was further found to correspond to the CB-stabilised simulated structure comparable to the one for I-based 2D systems (Fig. S13), which was found to be energetically comparable to the corresponding S–π-stabilised structure (Table S1), corroborating their co-existence in thin films.

Reference

[1] T. D. Kühne, M. Iannuzzi, M. Del Ben, V. V. Rybkin, P. Seewald, F. Stein, T. Laino, R. Z. Khaliullin, O. Schütt, F. Schiffmann, D. Golze, J. Wilhelm, S. Chulkov, M. H. Bani-Hashemian, V. Weber, U. Borštnik, M. Taillefumier, A. S. Jakobovits, A. Lazzaro, H. Pabst, T. Müller, R. Schade, M. Guidon, S. Andermatt, N. Holmberg, G. K. Schenter, A. Hehn, A. Bussy, F. Belleflamme, G. Tabacchi, A. Glöß, M. Lass, I. Bethune, C. J. Mundy, C. Plessl, M. Watkins, J. VandeVondele, M. Krack, J. Hutter, *J. Chem. Phys.* **2020**, *152*, 194103.

[2] S. Grimme, *J. Comp. Chem.* **2006**, *27*, 1787.

[3] S. Goedecker, M. Teter, J. Hutter, *Phys. Rev. B* **1996**, *54*, 1703.

[4] M. Krack, *Theor Chem Acc* **2005**, *114*, 145.

[5] J. VandeVondele, J. Hutter, *J. Chem. Phys.* **2007**, *127*, 114105.

[6] G. Bussi, D. Donadio, M. Parrinello, *J. Chem. Phys.* **2007**, *126*, 014101.

[7] J. Contreras-García, E. R. Johnson, S. Keinan, R. Chaudret, J.-P. Piquemal, D. N. Beratan, W. Yang, *J. Chem. Theory Comput.* **2011**, *7*, 625.

[8] S. Plimpton, *J. Comp. Phys.* **1995**, *117*, 1.

[9] W. C. Swope, H. C. Andersen, P. H. Berens, K. R. Wilson, *J. Chem. Phys.* **1982**, *76*, 637.

[10] S. Nosé, *J. Chem. Phys.* **1984**, *81*, 511.

[11] W. G. Hoover, *Phys. Rev. A* **1985**, *31*, 1695.

[12] P. Ahlawat, A. Hinderhofer, E. A. Alharbi, H. Lu, A. Ummadisingu, H. Niu, M. Invernizzi, S. M. Zakeeruddin, M. I. Dar, F. Schreiber, A. Hagfeldt, M. Grätzel, U. Rothlisberger, M. Parrinello, *Sci. Adv.* **2021**, *7*, eabe3326.

[13] L. S. Dodda, I. Cabeza de Vaca, J. Tirado-Rives, W. L. Jorgensen, *Nucleic Acids Res.* **2017**, *45*, W331.

[14] O. Buneman, *SIAM Rev.* **1983**, *25*, 425.

[15] O. H. Seeck, C. Deiter, K. Pflaum, F. Bertam, A. Beerlink, H. Franz, J. Horbach, H. Schulte-Schrepping, B. M. Murphy, M. Greve, O. Magnussen, *J. Synchrotron. Rad.* **2012**, *19*, 30.

[16] CrysAlis PRO. Rigaku Oxford Diffraction: Yarnton, Oxfordshire, England, 2017.

[17] G. M. Sheldrick, *Sect. A: Found. Crystallogr.* **2015**, A71, 3 – 8.

[18] G. M. Sheldrick, *Acta Crystallogr., Sect. C: Struct. Chem.* **2015**, *71*, 3– 8.

[19] (a) G. C. Fish; A. T. Terpstra; A. Ducinskas; M. Almalki; L. C. Carbone, L. Pfeifer, M. Graetzel, J.-E. Moser, J. Milic, *J. Phys. Chem. Lett.* **2023**, *14*, 6248. (b) W. R. Kitzmann, D. Hunger, A.-P. M. Reponen, C. Förster, R. Schoch, M. Bauer, S. Feldmann, J. van Slageren, K. Heinze, *Inorg. Chem.* **2023**, *62*, 15797.

[20] A. A. Sutanto, P. Caprioglio, N. Drigo, Y. J. Hofstetter, I. Garcia-Benito, V. I. E. Queloz, D. Neher, M. K. Nazeeruddin, M. Stolterfoht, Y. Vaynzof, G. Grancini, *Chem* **2021**, *7*, 1903.

[21] M. Axelsson, C. F. N. Marchiori, P. Huang, C. M. Araujo, H. Tian, *J. Am. Chem. Soc.* **2021**, *143*, 21229.

[22] Y. Li, J. V. Milić, A. Ummadisingu, J.-Y. Seo, J.-H. Im, H.-S. Kim, Y. Liu, M. I. Dar, S. M. Zakeeruddin, P. Wang, A. Hagfeldt, M. Grätzel, *Nano Lett.* **2018**, *19*, 150.

[23] J. V. Milić, J.-H. Im, D. J. Kubicki, A. Ummadisingu, J.-Y. Seo, Y. Li, M. A. Ruiz Preciado, M. I. Dar, S. M. Zakeeruddin, L. Emsley, M. Grätzel, *Adv. Energy Mater.* **2019**, *131*, 1900284.
